# Cortical morphology in patients with the deficit and non-deficit syndrome of schizophrenia: a worldwide meta- and mega-analyses

**DOI:** 10.1038/s41380-023-02221-w

**Published:** 2023-08-29

**Authors:** Nerisa Banaj, Daniela Vecchio, Fabrizio Piras, Pietro De Rossi, Juan Bustillo, Simone Ciufolini, Paola Dazzan, Marta Di Forti, Erin W. Dickie, Judith M. Ford, Paola Fuentes-Claramonte, Oliver Gruber, Amalia Guerrero-Pedraza, Holly K. Hamilton, Fleur M. Howells, Bernd Kraemer, Stephen M. Lawrie, Daniel H. Mathalon, Robin Murray, Edith Pomarol-Clotet, Steven G. Potkin, Adrian Preda, Joaquim Radua, Anja Richter, Raymond Salvador, Akira Sawa, Freda Scheffler, Kang Sim, Filip Spaniel, Dan J. Stein, Henk S. Temmingh, Sophia I. Thomopoulos, David Tomecek, Anne Uhlmann, Aristotle Voineskos, Kun Yang, Neda Jahanshad, Paul M. Thompson, Theo G. M. Van Erp, Jessica A. Turner, Gianfranco Spalletta, Federica Piras

**Affiliations:** 1 Neuropsychiatry Laboratory, Department of Clinical Neuroscience and Neurorehabilitation, IRCCS Santa Lucia Foundation, Rome, Italy; 2 Child and Adolescence Neuropsychiatry Unit, Bambino Gesù Children’s Hospital, IRCCS, Rome, Italy; 3 Psichiatry and Neuroscience, University of New Mexico, Albuquerque, NM USA; 4 Psychosis Studies, Institute of Psychiatry, Psychology and Neurology, King’s College London, London, UK; 5 Department of Psychological Medicine, Institute of Psychiatry, Psychology and Neurology, King’s College London, London, UK; 6 Center for Addiction and Mental Health, Campbell Family Mental Health Research Institute, Toronto, ON Canada; 7 Department of Psychiatry, University of Toronto, Toronto, ON Canada; 8 Kimel Family Lab, Centre for Addiction and Mental Health, Toronto, ON Canada; 9 San Francisco VA Health Care System, San Francisco, CA USA; 10 Department of Psychiatry and Behavioral Sciences, University of California, San Francisco, CA USA; 11 FIMDAG Sisters Hospitallers Research Foundation, Barcelona, Spain; 12 Biomedical Network Research Centre on Mental Health (CIBERSAM), Instituto de Salud Carlos III, Madrid, Spain; 13 Section for Experimental Psychopathology and Neuroimaging, Department of General Psychiatry, Heidelberg University, Heidelberg, Baden-Wuerttemberg Germany; 14 Hospital Benito Menni CASM, Barcelona, Spain; 15 Department of Psychiatry and Mental Health, University of Cape Town, Cape Town, Western Cape South Africa; 16 Division of Psychiatry, University of Edinburgh, Edinburg, EH10 5HF UK; 17 Department of Psychiatry, University of California Irvine, Newfoundland, NJ NJ 07435 USA; 18 Psychiatry and Human Behavior, University of California Irvine, Orange, CA 92868 USA; 19 Imaging of mood- and anxiety-related disorders (IMARD), Institut d’Investigacions Biomèdiques August Pi i Sunyer (IDIBAPS), Barcelona, 08036 Spain; 20 Medicina, University of Barcelona, Barcelona, 08036 Spain; 21 Department of Psychiatry, Johns Hopkins University School of Medicine, Baltimore, MD USA; 22 Department of Neuroscience, Johns Hopkins University School of Medicine, Baltimore, MD USA; 23 Department of Biomedical Engineering, Johns Hopkins University School of Medicine, Baltimore, MD USA; 24 Department of Genetic Medicine, Johns Hopkins University School of Medicine Baltimore, Baltimore, MD USA; 25 Department of Pharmacology, Johns Hopkins University School of Medicine, Baltimore, MD USA; 26 Department of Mental Health, Johns Hopkins Bloomberg School of Public Health, Baltimore, MD USA; 27 Department of Psychiatry and Mental Health, Neuroscience Institute, University of Cape Town, Cape Town, South Africa; 28 Brain Behavior Unit, Department of Psychiatry and Mental Health, University of Cape Town, Cape Town, South Africa; 29 West Region, Institute of Mental Health, National Healthcare Group, Singapore, Singapore; 30 Yong Loo Lin School of Medicine, National University of Singapore, Singapore, Singapore; 31 CARE, National Institute of Mental Health, Klecany, Czech Republic; 32 SAMRC Unit on Risk & Resilience in Mental Disorders, Department of Psychiatry & Neuroscience Institute, University of Cape Town, Cape Town, South Africa; 33 Department of Psychiatry and Mental Health, Valkenberg Psychiatric Hospital, Cape Town, Western Cape South Africa; 34 Imaging Genetics Center, Mark & Mary Stevens Neuroimaging and Informatics Institute, Keck School of Medicine, University of Southern California, Marina del Rey, CA USA; 35 Department of child and adolescent psychiatry, TU Dresden, Dresden, Saxony Germany; 36 Department of Psychiatry, University of Toronto, Temerty Faculty of Medicine, Toronto, ON Canada; 37 Clinical Translational Neuroscience Laboratory, Department of Psychiatry and Human Behavior, University of California Irvine, Irvine, CA USA; 38 Center for the Neurobiology of Learning and Memory, University of California Irvine, Irvine, CA USA; 39 Department of Psychiatry and Behavioral Health, Wexner Medical Center, The Ohio State University, Columbus, OH USA; 40 Menninger Department of Psychiatry and Behavioral Sciences, Baylor College of Medicine, Houston, TX USA

**Keywords:** Neuroscience, Schizophrenia

## Abstract

Converging evidence suggests that schizophrenia (SZ) with primary, enduring negative symptoms (i.e., Deficit SZ (DSZ)) represents a distinct entity within the SZ spectrum while the neurobiological underpinnings remain undetermined. In the largest dataset of DSZ and Non-Deficit (NDSZ), we conducted a meta-analysis of data from 1560 individuals (168 DSZ, 373 NDSZ, 1019 Healthy Controls (HC)) and a mega-analysis of a subsampled data from 944 individuals (115 DSZ, 254 NDSZ, 575 HC) collected across 9 worldwide research centers of the ENIGMA SZ Working Group (8 in the mega-analysis), to clarify whether they differ in terms of cortical morphology. In the meta-analysis, sites computed effect sizes for differences in cortical thickness and surface area between SZ and control groups using a harmonized pipeline. In the mega-analysis, cortical values of individuals with schizophrenia and control participants were analyzed across sites using mixed-model ANCOVAs. The meta-analysis of cortical thickness showed a converging pattern of widespread thinner cortex in fronto-parietal regions of the left hemisphere in both DSZ and NDSZ, when compared to HC. However, DSZ have more pronounced thickness abnormalities than NDSZ, mostly involving the right fronto-parietal cortices. As for surface area, NDSZ showed differences in fronto-parietal-temporo-occipital cortices as compared to HC, and in temporo-occipital cortices as compared to DSZ. Although DSZ and NDSZ show widespread overlapping regions of thinner cortex as compared to HC, cortical thinning seems to better typify DSZ, being more extensive and bilateral, while surface area alterations are more evident in NDSZ. Our findings demonstrate for the first time that DSZ and NDSZ are characterized by different neuroimaging phenotypes, supporting a nosological distinction between DSZ and NDSZ and point toward the separate disease hypothesis.

## Introduction

Unraveling schizophrenia (SZ) heterogeneity represents a formidable ongoing challenge enforcing the need to examine the neurobiological correlates of specific symptomatology. Since the current nosology likely reflects a group of diseases [1], discriminating between subtypes of schizophrenia characterized by more homogeneous enduring symptoms [1–3] may be an effective method for identifying unique neurobiological markers of specific disease entities within the SZ spectrum [4]. A clinically homogeneous subgroup of patients diagnosed with SZ showing primary, stable, and enduring negative symptoms (i.e., Deficit SZ (DSZ)) [1, 2] can be distinguished, representing up to one-third of individuals with SZ [5]. DSZ is characterized by persistent impairment and poorer long-term prognosis with a lower likelihood of recovery [6] compared to Non-Deficit SZ (NDSZ).

Clinical and neurobiological differences between DSZ and NDSZ patients [3, 7–12] can be identified at the first psychotic episode or even before clinical manifestation [3, 11, 13, 14]. Further evidence [1, 15, 16] suggests that these disease entities differ also in etiologic factors, course, and treatment response. Accordingly, several authors [1, 3, 17–20] support the hypothesis that DSZ could represent a separate disease within the SZ syndrome.

While the clinical manifestations of DSZ assume distinctive characteristics that have been well-described in prior works [8, 21–26], the neural substrates of the disorder are not sufficiently understood [27]. In fact, structural magnetic resonance imaging (MRI) studies have produced conflicting results. Although some studies reported white matter [4, 28] (WM) and gray matter [29–32] (GM) abnormalities in DSZ compared to NDSZ, others reported gray matter abnormalities in NDSZ patients only [33, 34], or no differences between the two patient subgroups [4].

Given such discordant results, a deeper insight may be gained by shifting the focus from volumetric measures to morphological parameters indexing different aspects of brain architecture. Indeed, cortical GM volume is defined as the product of two morphological indices (i.e., cortical thickness and surface area) and lower volume may reflect either thinner cortex, smaller surface area, or both. Although the exact neurobiological constituents remain unclear, surface area and cortical thickness may be determined by the number and the laminar patterning of cortical columns, respectively [35, 36], thus providing a biologically relevant decomposition of cortical volume. They show unique regional variations across the cerebral cortex, both at the regional and the whole-brain level, and have largely distinct genetic architectures [37–39]. Each of these morphometric features shows different age-related trajectories, thus providing diverse information on brain development, and may offer more fundamental insights than comparisons of GM volumes [37, 38] particularly in disorders characterized by neurodevelopmental disturbances [40].

Prior structural neuroimaging studies have found that cortical thickness and surface area are abnormal in SZ patients as a whole [41, 42], and these abnormalities correlated with negative symptoms. However, to date, only a few studies have explored such indices in DSZ [4, 31, 43], reporting conflicting findings. Specifically, one study observed thinner cortex in the anterior cingulate [31] and temporo-parietal junction areas in DSZ compared to NDSZ [43], while another study observed no differences between the two groups [4]. Surface area findings are more homogeneous, suggesting no differences between DSZ and NDSZ [4, 43]. Nevertheless, limited power, based on sample sizes ranging from 18 to 40 subjects diagnosed with DSZ, may underlie these inconsistent or negative results. Further, methodological differences, with some studies examining vertex-based [4, 43] and others taking region of interest (ROI) approaches [31], and differences in data acquisition and processing protocols may have further contributed to the observed discrepancies in findings. To overcome the heterogeneity in image processing and to increase sample sizes, brain imaging consortia offer the opportunity to bring together data from all over the world to achieve higher statistical power using standardized processing and analysis methods. Here, we gathered data from several worldwide research centers contributing to the ENIGMA SZ working group to create the largest data set of DSZ and NDSZ examined to date.

We compared cortical thickness and surface area measures to test whether patients with DSZ differ from patients with NDSZ in morphological parameters indexing different aspects of brain architecture. Since both meta- and mega-analysis approaches have advantages and disadvantages (see [44–46] for reviews), both methods were adopted to investigate whether the mega-analytic design could achieve greater sensitivity in detecting more subtle brain abnormalities owing to a greater information preservation. This is in line with previous ENIGMA studies [47–49].

We hypothesized that both SZ subgroups would exhibit widespread cortical morphometric abnormalities, compared to HC. In addition, we hypothesized differential patterns of cortical thickness and surface area anomalies in DSZ and NDSZ. Identifying overlapping and divergent morphological features in SZ subtypes may provide important hints for delineating common and unique pathological pathways within the SZ syndrome, which is critical for improving diagnostic and therapeutic accuracy in this heterogeneous disorder.

## Materials and methods

### Study sample

The current study included patients diagnosed with SZ and HC. Diagnosis was based on the Diagnostic and Statistical Manual of Mental Disorders (DSM, editions IV-TR [50] or 5 [51]) or the International Classification of Diseases (ICD-10) [52] criteria using the related version of the Structured Clinical Interview for DSM Disorders (SCID) [53, 54], and/or a review of case files/medical records by trained clinicians (see Supplementary Table S1 for more details). All individuals had the Positive and Negative Syndrome Scale [55] (PANSS) ratings and T1-weighted structural brain MRI data available. HC were recruited and screened for a current or lifetime history of psychiatric disorders using the SCID (see Supplementary Table S1 for more details).

According to the availability and ethical permission of each research site to share individual raw data or between-group effect size data, we analyzed two different samples for meta- and mega-analysis. The meta-analysis sample comprised 9 cohorts from 8 countries totaling 1560 participants, including 1019 HC, 168 DSZ and 373 NDSZ. The mega-analysis sample included data from 8 cohorts in 7 countries (1 site did not have permission to share individual data), comprising 944 participants (i.e., 575 HC, 115 DSZ and 254 NDSZ). In the mega-analysis the DSZ, NDSZ and HC participants were one-to-one age-matched ( ± 2 years) across-sex by an investigator blinded to study aims in order to reduce confounding effects of age-dependent changes in brain morphology [56]. Participants’ demographics (i.e., age and sex) were collected for both the meta- and mega-analyses samples, while clinical data (i.e., stable dosage of chlorpromazine equivalents of antipsychotic treatments, duration of illness, rates of positive, negative and general symptoms) were collected only for the mega-analysis.

Each study sample was collected with participants’ written informed consent approved by local Institutional Review Boards. The authors declared that all procedures contributing to this work comply with the ethical standards of the relevant national and institutional committees on human experimentation and with the Helsinki Declaration of 1975, as revised in 2008. No subject identifying data were shared among the ENIGMA institutions.

### Deficit/Non-deficit classification

The characterization of DSZ was performed according to the proxy case identification method (i.e., the proxy for the deficit syndrome (PDS) [57]) using PANSS [55]. The PDS has good specificity, sensitivity, and accuracy [57]. Furthermore, the PDS has been repeatedly shown to be a valid tool for the categorization of patients into DSZ and NDSZ, in both early-episode and chronic populations [58, 59]. Specifically, the PDS score is defined as a composite score - that is, the sum of the scores (from the PANSS) of the anxiety, guilt feelings, depressive mood and hostility items subtracted from the score for blunted affect item score. A cut-off of 2 was used to classify DSZ and NDSZ [57]. This calculation reflects primary and persistent negative symptoms in the deficit syndrome [2]. To enhance the likelihood of correct classification, and reduce potential false positives [60], only patients who ranked in the top and bottom quartiles of PDS scores were defined as having DSZ and NDSZ, respectively. Hence, we eliminated the middle quartile of patients from the SZ sample, which can be considered a highly mixed group of DSZ and NDSZ patients, while the inclusion of the two extreme quartiles assured the selection of relatively “pure” groups of individuals showing distinctive clinical symptoms. This type of relatively conservative categorization method has already been employed in previous studies [12, 61]. The entire categorization process was managed by the coordinating site on the data shared by each research site and then redistributed for in situ pre-processing of neuroimaging data.

### Image acquisition and processing

Structural T1-weighted brain scans were acquired and processed locally at each research site for DSZ, NDSZ, and HC. Scanner and acquisition details at each site are provided in Table S1 in the online supplement. All sites processed the T1-weighted structural brain scans for each participant using an automated and validated pipeline, i.e., “recon-all” as implemented in FreeSurfer (https://surfer.nmr.mgh.harvard.edu/). Specifically, the averages of cortical thickness and surface area were extracted for each of the 70 cortical regions of interest (ROI) (34 regions per hemisphere + 1 whole hemisphere) based on the Desikan-Killiany parcellation scheme [62], as well as total brain surface area and mean cortical thickness. The use of FreeSurfer in multisite analyses has been validated in previous ENIGMA studies [47, 63]. As a final step, the pipeline ended with the visual inspection of all data in a series of standard planes so as to detect potential outliers (http://enigma.ini.usc.edu/protocols/imaging-protocols/).

### Statistical analysis

#### Meta-analysis

In order to calculate Cohen’s *d* effect sizes for the meta-analysis, each site examined the differences between DSZ and NDSZ (separately) and HC in cortical thickness and surface area measures using an ANCOVA model. Neuroanatomical measures of each ROI entered the model as the dependent variable and a binary indicator of diagnosis (0 = control, 1 = case) as the predictor of interest. The model included intracranial volume (ICV), age, age^2^, sex, age-by-sex interaction, and age^2^-by-sex interaction as covariates. Age and age^2^ were included to adjust for linear and non-linear effects of age on brain structure [64]. To obtain a standardized difference in means, the *t*-statistic of the diagnosis variable obtained from regression models was used to compute the Cohen’s *d* effect size metrics.

All regression models and effect size estimates were computed individually at each site and a random-effects inverse-variance weighted meta-analysis was conducted at the coordinating site (the Laboratory of Neuropsychiatry at the Santa Lucia Foundation IRCCS in Rome, Italy) using Comprehensive Meta-Analysis (CMA) software, version 2 [65]. Specifically, the coordinating site was the one that collected and analyzed the effect sizes as computed from each participating site. Random-effect meta-analyses were performed for both DSZ and NDSZ separately. The Q statistic, *I*^2^ and τ^2^ scores were computed to determine the total heterogeneity of the effect sizes and the between-site variance. The stability of the overall effect size estimate was tested using a ‘leave one out’ sensitivity analysis, to assess whether results were dependent on site-specific confounding effects. Specifically, sensitivity analysis shows how the overall effects size changes when one dataset at a time is removed and gives insight into between-site variability and sampling error.

A mixed effects subgroups analysis was also performed to directly compare effect size estimates between the DSZ and NDSZ groups. Specifically, in this subgroup analysis the variance across sites was assumed to be randomly influenced by factors inherent to dataset characteristics (DSZ, NDSZ) plus sampling error, while the effect size is expected to be equivalent and fixed in datasets sampled from the same population.

Demographic differences among groups were assessed using ANOVA or chi-squared tests.

Bonferroni correction of multiple comparisons was applied.

#### Mega-analysis

The mega-analysis was performed by pooling all individual subject cortical thickness and surface measures from 8 sites; 1 site did not have permission to share this data.

Group differences in demographics and clinical characteristics were assessed using ANOVA, Student’s *t* or chi-squared tests.

Brain morphometry differences among DSZ_,_ NDSZ and HC were evaluated using univariate mixed-effect ANCOVA models. Specifically, cortical thickness or surface measures of each ROI were included in the model as the dependent variables. Group (DSZ, NDSZ, HC) was entered in the model as a fixed factor, and site as a random factor. Similar to the meta-analyses, age, age^2^, sex, age-by-sex interaction, and age^2^-by-sex interaction were added as additional confounding covariates to account for influences of demographic factors on anatomical inter-individual variability. All statistics were performed using SPSS Statistics version 25.0 (IBM, Armonk, N.Y.) considering *p* < 0.05 as the statistical threshold for significance. Bonferroni corrected post hoc comparisons were performed for significant results from the thickness and surface mega-analyses.

## Results

### Meta-analysis

HC participants included in the meta-analysis were significantly younger than SZ, though there were no significant age differences between the DSZ and NDSZ groups. Sex distribution also differed between the three groups included in the meta-analyses (Table 1).

**Table 1:** Demographical information for HC, DSZ and NDSZ in Mega and Meta-Analysis samples; Clinical information for DSZ and NDSZ patients in the Mega-Analysis sample.

	HC	DSZ	ND-SZ	t, F or χ2	df	*p*
*Meta-Analysis*						
Age (years), Mean (SD)	33.51 (13)	37.73 (13.1)	37.2 (12.2)	16.17	21561	<0.001*
Males, *n* (%)	546 (54)	110 (67)	234 (62)	15.64	2	<0.001*
*Mega-Analysis*						
Age (years), Mean (SD)	34.5 (12.5)	36.5 (12.5)	35.7 (11.6)	1.74	2941	0.175
Males, *n* (%)	272 (43)	93 (80)	160 (63)	51.4	2	<0.001*
Duration of illness (Years), Mean (SD)	–	13.8 (12.6)	11.9 (11.7)	1.09	1220	0.298
Chlorpromazine equivalents, Mean (SD)	–	404.5 (414.4)	390.8 (454.2)	0.055	1266	0.814
PANSS_Pos, Mean (SD)	–	13.6 (5.2)	18 (6.9)	36.7	1367	<0.001*
PANSS_Neg, Mean (SD)	–	21 (7.5)	16.2 (7)	34.7	1367	<0.001*
PANSS_Gen, Mean (SD)	–	29.7 (10)	37.5 (12.6)	33.8	1367	<0.001*

*df* degrees of freedom, *SD* standard deviation, *PANSS_Pos* positive symptoms subscale from Positive and Negative Symptoms Scale, *PANSS_Neg* negative symptoms subscale from Positive and Negative Symptoms Scale, *PANSS_Gen* general symptoms subscale from Positive and Negative Symptoms Scale, Type of antipsychotic Treatment: *T* Typical, *A* Atypical, *B* Both, *N* Nothing.

^*^Statistically significant differences at *p* < 0.05.

### Cortical thickness

The meta-analysis of cortical thickness found 53 ROIs (out of the 70 analyzed) with significantly thinner cortex in both DSZ and NDSZ, compared to HC. However, compared to HC, the left lateral orbito-frontal, the rostral part of the left anterior cingulate, and the right transverse temporal cortex were significantly thinner in the DSZ group only, while the left lateral-occipital and lingual cortex were significantly thinner in NDSZ only (see Supplementary Tables S7 and S8).

Significant heterogeneity was observed in 18 ROIs for the DSZ and 36 ROIs for the NDSZ, based on the moderate amount of between-site variance (*I*^*2*^ > 50%; Supplementary Tables S7 and S8), suggesting that variability in the study population characteristics was higher in the NDSZ group. Moreover, for seven additional effects, the sensitivity analysis (data available upon request) revealed that the removal of individual datasets (from 1 to 8) impacted model significance.

Considering only homogenous (consistent across sites, non-significant and low heterogeneity between datasets; *I*^*2*^ < 50%) and robust (surviving sensitivity analysis and Bonferroni correction for multiple comparisons) effects, a thinner cortex was observable in 9 overlapping ROIs for both SZ groups, in 10 ROIs for DSZ only (mostly in the right hemisphere) and 2 ROIs for NDSZ only (Table 2).

**Table 2:** Cortical thickness results from random-effect Meta-Analysis.

Cortical regions	Groups	Effect size and 95% confidence interval						Test of null (2-Tail)		Heterogeneity				Tau- Sqrd
		*N*	Point estim	Std err	Var	Low limit	Up limit	*Z*-value	*P value*	*Q* value	df	*P value*	I- sqrd	
Cortical regions of commonly reduced thickness in DSZ and NDSZ														
L. Caudal middle frontal	DSZ	9	−0.59	0.11	0.01	−0.80	−0.37	−5.37	<0.0001	11.12	8	0.1948	28.08	0.03
	NDSZ	9	−0.41	0.06	0.00	−0.54	−0.29	−6.46	<0.0001	6.68	8	0.5712	0.00	0.00
L. Inferior parietal	DSZ	9	−0.48	0.09	0.01	−0.67	−0.30	−5.14	<0.0001	8.89	8	0.3516	10.02	0.01
	NDSZ	9	−0.35	0.08	0.01	−0.50	−0.19	−4.42	<0.0001	10.96	8	0.2040	27.00	0.01
L. Pars opercularis	DSZ	9	−0.57	0.13	0.02	−0.83	−0.31	−4.30	<0.0001	15.51	8	0.0500	48.40	0.07
	NDSZ	9	−0.39	0.07	0.00	−0.52	−0.26	−5.72	<0.0001	8.80	8	0.3595	9.09	0.00
L. Postcentral	DSZ	9	−0.49	0.12	0.01	−0.73	−0.25	−4.03	<0.0001	13.37	8	0.0999	40.14	0.05
	NDSZ	9	−0.34	0.09	0.01	−0.52	−0.16	−3.63	0.0003	14.91	8	0.0609	46.35	0.03
L. Precentral	DSZ	9	−0.54	0.13	0.02	−0.80	−0.29	−4.19	<0.0001	15.05	8	0.0582	46.85	0.06
	NDSZ	9	−0.33	0.09	0.01	−0.50	−0.15	−3.71	0.0002	13.37	8	0.0997	40.18	0.03
L. Supramarginal	DSZ	9	−0.57	0.09	0.01	−0.74	−0.40	−6.54	<0.0001	5.64	8	0.6870	0.00	0.00
	NDSZ	9	−0.37	0.06	0.00	−0.49	−0.24	−5.77	<0.0001	6.77	8	0.5617	0.00	0.00
R. Bankssts	DSZ	9	−0.53	0.11	0.01	−0.75	−0.32	−4.82	<0.0001	11.20	8	0.1907	28.56	0.02
	NDSZ	9	−0.38	0.08	0.01	−0.54	−0.21	−4.50	<0.0001	12.31	8	0.1379	35.01	0.02
R. Caudal middle frontal	DSZ	9	−0.47	0.09	0.01	−0.64	−0.31	−5.51	<0.0001	4.89	8	0.7688	0.00	0.02
	NDSZ	9	−0.32	0.08	0.01	−0.48	−0.16	−3.91	<0.0001	11.76	8	0.1621	32.00	0.02
R. Parahippocampal	DSZ	9	−0.42	0.11	0.01	−0.62	−0.21	−3.96	<0.0001	10.50	8	0.2319	23.79	0.03
	NDSZ	9	−0.32	0.09	0.01	−0.49	−0.15	−3.68	0.0002	13.19	8	0.1056	39.33	0.03
Cortical regions of reduced thickness in DSZ														
L. Banks sts	DSZ	9	−0.46	0.11	0.01	−0.68	−0.24	−4.08	<0.0001	11.17	8	0.1924	28.36	0.03
L. Superior parietal	DSZ	9	−0.33	0.09	0.01	−0.50	−0.16	−3.87	0.0001	3.86	8	0.8693	0.00	0.00
R. Inferior parietal	DSZ	9	−0.49	0.11	0.01	−0.71	−0.27	−4.34	<0.0001	11.73	8	0.1637	31.79	0.05
R. Pars orbitalis	DSZ	9	−0.42	0.10	0.01	−0.62	−0.22	−4.14	<0.0001	9.83	8	0.2773	18.60	0.04
R. Pars triangularis	DSZ	9	−0.39	0.10	0.01	−0.59	−0.19	−3.89	0.0001	9.83	8	0.2772	18.61	0.02
R. Precentral	DSZ	9	−0.46	0.12	0.01	−0.69	−0.23	−3.95	<0.0001	12.34	8	0.1366	35.17	0.05
R. Precuneus	DSZ	9	−0.40	0.10	0.01	−0.59	−0.21	−4.07	<0.0001	9.41	8	0.3087	15.01	0.05
R. Superior frontal	DSZ	9	−0.58	0.13	0.02	−0.84	−0.32	−4.41	<0.0001	15.48	8	0.0505	48.32	0.05
R. Superior parietal	DSZ	9	−0.33	0.09	0.01	−0.50	−0.16	−3.86	0.0001	6.09	8	0.6366	0.00	0.01
R. Supramarginal	DSZ	9	−0.60	0.13	0.02	−0.85	−0.34	−4.58	<0.0001	14.98	8	0.0596	46.58	0.04
Cortical regions of reduced thickness in NDSZ														
L. Frontal pole	NDSZ	9	−0.27	0.07	0.00	−0.40	−0.14	−3.98	<0.0001	8.61	8	0.3764	7.06	0.00
R. Paracentral	NDSZ	9	−0.25	0.07	0.00	−0.38	−0.12	−3.74	0.0002	8.66	8	0.3720	7.59	0.00

Only robust (i.e., surviving sensitivity analysis and Bonferroni correction for multiple comparisons) are reported.

*df* degrees of freedom, *L.* left, *Point estim* estimated standard difference, *R.* right, *Sqrd* Squared, *Std err* Standard Error, *sts* superior temporal sulcus, *Var* Variance.

**Fig. 1:**
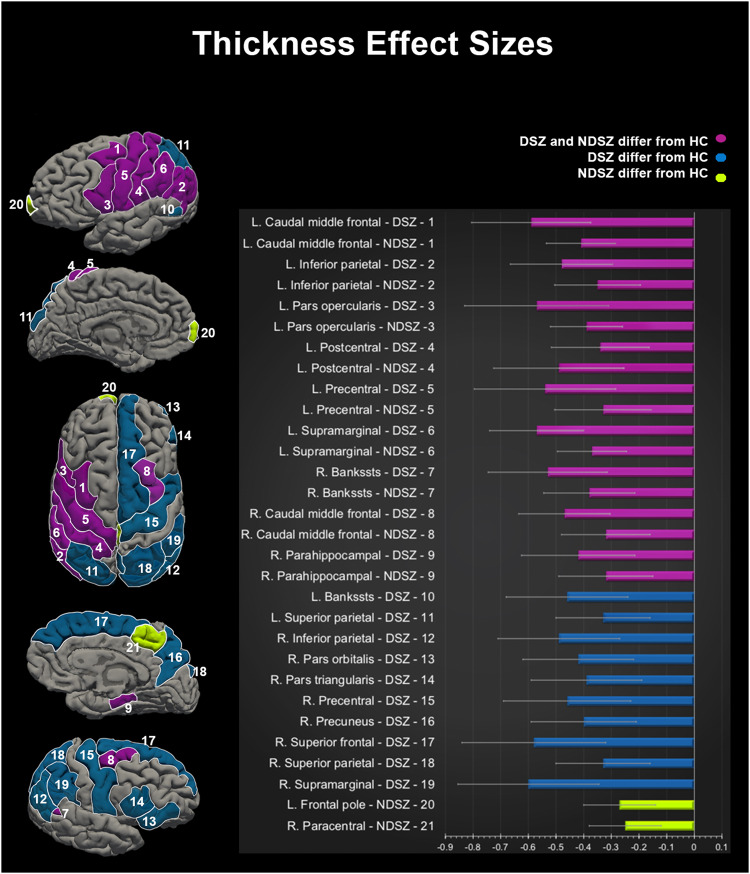
Results of the random-effect meta-analysis on cortical thickness. Cohen’s d effect sizes comparing Deficit and Non-Deficit Schizophrenia samples to Healthy Controls. L. Left Hemisphere, R. Right Hemisphere, DSZ Deficit Schizophrenia group, NDSZ Non-Deficit Schizophrenia group, HC Healthy Controls group, Histogram bars represent Cohen’s d effect sizes after meta-analysis; error bars represent 95% confidence interval. Blue bars represent regions in which DSZ significantly differ from HC only; purple bars represent regions in which either DSZ or NDSZ significantly differ from HC; lime yellow bars represent regions in which NDSZ significantly differ from HC only.

The meta-analytic subgroup analysis on cortical thickness showed no significant differences in effect sizes between DSZ and NDSZ (Supplementary Tables S7 and S8; Fig. 1).

### Surface area

The meta-analysis on cortical surface area (see Supplementary Tables S9 and S10) showed that in comparison to HC, DSZ had a smaller surface area in the left fusiform gyrus, the left superior frontal, and the left pars triangularis regions. Results for the latter two ROIs were not robust, since they did not survive sensitivity analysis, while the effect in the left fusiform gyrus did not survive to Bonferroni correction. NDSZ showed a significantly smaller cortical surface area in the caudal part of the bilateral anterior cingulate cortex, the right isthmus of the cingulate cortex and the right precentral cortex. Only results regarding the caudal part of the right anterior cingulate cortex were consistent and robust, although they did not survive to Bonferroni correction, while—for the remaining ROIs—model significance was impacted by the removal of individual datasets (results available upon request).

The meta-analytic subgroup analysis on cortical surface showed no differences in effect sizes between DSZ and NDSZ (Supplementary Tables S9 and S10).

### Mega-analysis

A significantly different sex distribution was observed in the three groups included in the mega-analyses, while as expected owing to the matching procedure adopted, no age difference was found (Table 1). Congruently with the diagnostic phenotype, ratings of positive, negative and general psychopathology symptoms were different in the DSZ and NDSZ groups (Table 1). No significant differences were observed for duration of illness or pharmacological treatment dosages (chlorpromazine equivalents).

### Cortical thickness

Mixed-model ANCOVAs on cortical thickness measures from 70 ROIs revealed a significant effect of diagnosis (Table 3) in the right isthmus of the cingulate cortex and the right banks of the superior temporal sulcus. Bonferroni corrected post hoc comparisons showed that, compared to HC, the DSZ group had a thinner right isthmus, while NDSZ had thinner right banks of the superior temporal sulcus. Moreover, measures for left and right mean thickness showed a significant group effect with lower thickness in both DSZ and NDSZ when compared to HC. No significant differences were found between DSZ and NDSZ.

**Table 3:** ANCOVA and post hoc results from Mega-Analyses of cortical surface and thickness measures.

Cortical regions	ANCOVA			Bonferroni Corrected Post Hoc					
				DSZ vs HC		NDSZ vs HC		DSZ vs NDSZ	
	F	df	*p*	Mean diff	*p*_corr_	Mean diff	*p*_corr_	Mean diff	*p*_corr_
*Thickness*									
R. Banks sts	4.86	2906	0.01*	−0.027	0.6	−0.048	0.012	0.021	1
R. Isthmus cingulate	4.66	2910	0.02*	−0.086	0.003*	−0.039	0.187	−0.048	0.291
L. Thickness	16.74	2911	<0.0001*	−0.075	<0.0001*	−0.066	<0.0001*	−0.008	1
R. Thickness	17.95	2911	<0.0001*	−0.071	<0.0001*	−0.062	<0.0001*	−0.008	1
*Surface*									
L. Lateral occipital	5.4	2906	0.01*	−23.17	1	−198.13	<0.0001*	174.96	0.02*
L. Precuneus	3.59	2910	0.04*	−6.67	1	84.01	0.02*	−90.68	0.13
L. Supramarginal	5.23	2910	0.01*	64.94	0.72	142.04	0.003*	−77.1	0.65
R. Caudal anterior cingulate	3.76	2911	0.03*	−31.4	0.27	−36.87	0.04*	5.47	1
R. Inferior temporal	3.92	2909	0.04*	−15.13	1	−156.56	<0.0001*	141.43	0.005*
R. Lateral occipital	4.93	2909	0.02*	−101.51	0.37	−359.27	<0.0001*	257.76	0.0004*
R. Lingual	3.99	2908	0.04*	−27.25	1	−228.95	<0.0001*	201.7	<0.0001*
R. Pericalcarine	4.16	2906	0.03*	−18.49	1	−132.45	<0.0001*	113.96	0.002*
R. Superior frontal	3.31	2911	0.048*	108.58	0.3	132.95	0.034*	−24.37	1

*df* degrees of freedom, *L.* left, *R.* right, *sts* superior temporal sulcus.

^*^Statistically significant differences at *p* < 0.05.

### Surface area

Mixed-model ANCOVAs on surface area measures showed a significant effect of diagnosis in the lateral occipital cortices (bilaterally), the left precuneus and supramarginal gyrus, the right caudal part of the anterior cingulate, pericalcarine, inferior temporal, lingual and superior frontal cortices (Table 3).

**Fig. 2:**
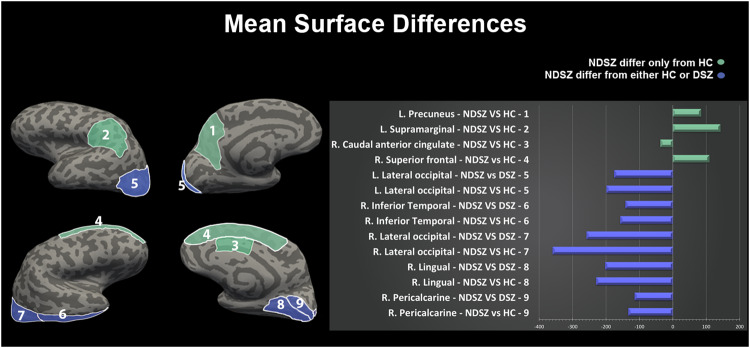
Bonferroni corrected post hoc comparisons from the mega-analysis on surface area. Mean differences comparing Deficit and Non-Deficit Schizophrenia samples to Healthy Controls. L. Left Hemisphere, R. Right Hemisphere, DSZ Deficit Schizophrenia group, NDSZ Non-Deficit Schizophrenia group, HC Healthy Controls group, Histogram represents the mean differences in each surface area comparison. Green bars represent regions in which NDSZ significantly differ from HC only; violet bars represent regions in which NDSZ significantly differ from either HC or DSZ.

Bonferroni corrected post hoc comparisons showed that the NDSZ group had a smaller surface area in the bilateral lateral occipital cortices (more pronounced in the right hemisphere), the right pericalcarine, inferior temporal and lingual cortices, as compared to both HC and DSZ. Further, NDSZ exhibited a smaller surface area in the caudal part of the right anterior cingulate cortex and larger surface area in the left precuneus, the left supramarginal gyrus and the right superior frontal cortex, as compared to HC (Table 3; Fig. 2).

## Discussion

In this largest-ever coordinated neuroimaging study conducted on morphological brain measures in DSZ and NDSZ we found different results from meta- and mega-analyses, coherently with the methodological differences existing between the two. Indeed, while the meta-analysis can be considered the most powerful approach here, due to the largest sample size included, the mega-analysis should be regarded as the most controlled one, due to the additional matching procedure. Therefore, we have taken into account the results of both analyses as mutually reinforcing, thereby demonstrating that: (1) both DSZ and NDSZ have cortical thickness abnormalities, but only NDSZ have surface abnormalities, as compared to HC; (2) DSZ have more pronounced thickness abnormalities than NDSZ, mostly involving the right hemisphere; (3) NDSZ have smaller temporo-occipital surface area than DSZ.

Several evidences suggest that cortical thickness and surface area are modulated by largely independent genetic factors, being also phenotypically unrelated [37, 38], and are associated with different cellular processes within the cerebral cortex [36, 66]. Indeed, according to the radial unit hypothesis, surface area is primarily driven by the number of cerebral cortical columns, and cortical thickness is mostly determined by the number and size of cells within a column (*ibidem*). Moreover, cortical thickness and surface area differ one from another in their developmental trajectories [67], maturation timing and they result from different ontogenic stages during corticogenesis [68]. Indeed, while the former seems to be more susceptible to environmental factors [69], the latter may be more influenced by early neurodevelopmental and genetic factors [37, 38]. Few and inconsistent studies have examined the genetics of DSZ and NDSZ (see [18] for a review), and future longitudinal studies will shed light on potential differences in abnormal developmental trajectories of cortical thickness and surface area between the two. However, our findings demonstrate for the first time that DSZ and NDSZ are characterized by different neuroimaging phenotypes, being DSZ typified by more pronounced abnormalities in the number (or size) of cortical neurons, while NDSZ in the number of cortical columns.

Apart from this divergence, and in line with previous morphological data of widespread cortical thinning in SZ [41, 42], our findings also bring new evidence on shared cortical thickness changes in DSZ and NDSZ, mostly in left fronto-parietal regions.

Abnormal functioning of the parietal lobe was previously reported in SZ [70] and associated with psychotic-like experiences [71], which would specifically affect cortical thickness as regional variations in parietal gray matter are particularly shaped by environmental factors even in normal developing adolescents [72]. Moreover, parietal cortices are known to participate in different neuropsychological functions that are affected in SZ patients [73]. Specifically, they play a crucial role in the storage and retrieval of verbal information, providing support to the frontal lobe [74]. In addition, the parietal lobes are responsible for significant processing related to spatial perception and attention [75–77], and activation of parietal regions along with prefrontal and medial temporal lobes are necessary to successfully encode episodic memories [78]. Interestingly, these regions are also involved in awareness-related processes [79, 80], and poor clinical and cognitive insight are key psychopathological features of SZ that worsen patients’ psychosocial functioning, clinical outcomes and treatment adherence [79–84]. Our results of thinner parietal and frontal cortices in either DSZ and NDSZ, are in line with previous imaging studies suggesting that in SZ gray matter abnormalities start earlier in parietal lobes and proceed to frontal regions (see [70] for a review). However, our findings clearly highlight a different fronto-parietal involvement in the two SZ subgroups, being more extensive and bilateral in the DSZ only.

Specifically, while thickness abnormalities shared by both SZ subgroups were mainly in the left hemisphere, DSZ showed extensive thickness abnormalities in the right hemisphere. Neuroscientific literature and neuropsychological evidence support an overall right hemispheric dominance for emotion, attention, and arousal [85]. Moreover, the right hemisphere modulates higher order language functions, essential for an accurate understanding of someone’s communicative intent [86]. Impairments in correctly inferring other people’s communicative intents, as well as deficits in processing emotional expressions and perceiving emotional intensity, also mediated by the right hemisphere [87], could significantly contribute to the social interaction deficits that are characteristic of DSZ [88].

Our findings also reveal that NDSZ demonstrate a distinctive decrease in cortical thickness of both the left frontal pole and the right paracentral lobule. A recent review [89] suggested that the frontal pole is significantly affected by the pathophysiology of SZ, with relevant alterations in the many high-order cognitive functions subtended by this region like emotion, memory, executive functions, and cognitive conflict resolution (*ibidem*). Regarding the paracentral lobule, which is functionally interconnected with other frontal and parietal regions [90], previous studies suggested that owing its involvement in motor and spatial attention functions, some motor abnormalities (e.g., gesture deficits and neurological soft signs) and attentional impairments observable in SZ could be consequent to structural abnormalities in this area, and associated with impaired psychosocial functioning.

Cortical functions mediated by either the frontal pole and the paracentral lobule seem to be related to the negative symptomatology, rather than to the positive symptoms characterizing NDSZ. However, negative symptoms are often present also in this SZ subgroup, even if less severe and persistent. In accordance with our findings, it is possible to contend that the superior psychosocial adaptation exhibited by individuals with fewer negative symptoms [91] might be due, in part, to the relatively preserved anatomical integrity of the right parietal lobe, despite the observed reduction in cortical thickness within the frontal pole and the paracentral lobule. In addition, the greater prevalence of thickness abnormalities in the right hemisphere of the DSZ subgroup perfectly aligns with the characteristic symptomatology of the diagnosis, which entails deficits in social interaction, withdrawal, and emotional blunting.

However, it is worth noting that despite the reported distinct cortical correlates of symptomatology, no significant results were found in the direct comparison of DSZ and NDSZ groups, neither in the meta- nor mega-analysis. To the best of our knowledge, only one study [43] has reported thinner cortices in DSZ as directly compared to NDSZ in some regions (i.e., bilateral anterior cingulate and left temporo-parietal junction area). Methodological differences and sampling biases, with patient groups predominantly—or exclusively—consisting of male individuals, may explain such discrepant results.

Contrariwise, when subgroups were directly compared in the mega-analysis on surface area, NDSZ showed a specific pattern of surface alterations, both in the comparison with DSZ and HC, demonstrating for the first time, that smaller surface area in temporo-occipital regions may be characteristic of this subgroup. Prior studies have suggested that abnormalities in temporo-occipital regions may play a central role in SZ psychopathology and in particular, in the development of positive symptoms such as delusions and hallucinations [92–95]. Consistent with the deficit/non-deficit concept [1, 61], the NDSZ patients here analyzed had more severe positive symptoms than the DSZ patients. Consequently, atypical patterns of cortical surface area may be associated with the clinical manifestations of positive symptoms, which are prevalent in this particular subtype of schizophrenia.

Additional results from the analyses of surface measures showed that the NDSZ group exhibited larger surface areas in the fronto-parietal cortices and smaller surface area in the right caudal anterior cingulate cortex, as compared to HC. Since a higher degree of cortical gyrification in the parietal lobe—an index intrinsically related to surface area measures [96]—has been related to positive symptoms severity in NDSZ only [27], it is reasonable to assume that neurodevelopmental processes related to cortical expansion and folding are involved in the pathophysiology of this SZ sub-type. Likewise, the smaller surface area of the right caudal anterior cingulate in NDSZ—a region involved in cognitive control processes [97, 98]—may be linked to the persistence of positive symptoms [99], since dysfunctional cognitive control is crucial for hallucinations and delusions.

The results of this study have to be interpreted in light of the following potential limitations. First, the deficit/non-deficit classification is based on a proxy case identification method (i.e., PDS [57]), while the gold standard for the documentation of the deficit syndrome relies on a semi-structured interview [100]. However, a large number of studies have employed the PDS [4, 6, 12, 21, 101, 102], and others have already demonstrated that PDS is both reliable [57, 58] and consistent [6, 61] in diagnosing DSZ. Second, given the cross-sectional design of this study, we couldn’t directly examine the duration of deficit-like features, which is one of the criteria for the deficit syndrome [57]. However, DSZ patients defined by the PDS score were characterized by more severe negative symptoms, but not by more severe positive symptoms, thus suggesting that heightened negative symptoms in DSZ are not a secondary result of heightened positive symptoms. Another aspect to consider when interpreting the results is that we used existing data across samples worldwide, with research sites using different scanners and imaging acquisition protocols. Thus, we cannot fully exclude the potential influence of these measurement protocols on the data. However, we included site as a random factor in the analyses in order to statistically control for scanner effects. In addition, our strategy of ensuring great methodological homogeneity by standardizing brain segmentation techniques and statistical models across all participating samples, increased the power in detecting even small effects. This method generates highly significant findings and allows for systematic investigation of the effects of clinical characteristics on brain alterations in psychiatric patients. A similar strategy has been used in parallel by other ENIGMA working groups [47, 63, 103].

Finally, another potential limitation may stem from the different samples in the mega- and meta-analyses as one of the included sites did not have the ethical permission to share individual subject data. Nevertheless, the mega-analytic approach, which preserves more information—allowed us to map, for the first time, cortical surface differences between DSZ and NDSZ subgroups.

In summary, the results of our study suggest that both DSZ and NDSZ exhibit shared and distinct morphological anomalies, including a widespread thinner cortex, which may contribute to differences in symptomatology between the two subtypes. Notably, a specific pattern of thinner cortex in bilateral fronto-parietal cortices appears to be associated with primary negative symptoms, while altered cortical surface area in fronto-parietal and occipital regions may distinguish vulnerability to prominent positive symptoms. These findings provide empirical support for the nosological differentiation of DSZ and NDSZ, and suggest the existence of distinct subtypes of the disease characterized by unique neuroimaging phenotypes, possibly subtended by different genetic/environmental effects on the developing brain. Dissociating symptomatic and anatomical subtypes in heterogenous syndromes like SZ, is critical for improving the clinical practice of goal-directed and personalized treatments.

### Supplementary information

Supplementary information

## Data Availability

The deidentified summary data and code that support the findings of this study are available upon reasonable request from the corresponding author. The data are not all publicly available in a repository as they may contain information that could compromise the privacy of research participants. There are also data sharing restrictions imposed by some of the (i) ethical review boards of the participating sites, and consent documents; (ii) national and trans-national data sharing laws; and (iii) institutional processes, some of which require a signed DTA for limited and predefined data use. However, we welcome sharing data with researchers, requiring that they become members of the ENIGMA Schizophrenia working group and submit an analysis plan for a secondary project for group review. Once this analysis plan is approved, access to the relevant data will be provided contingent on data availability, local PI approval and compliance with all supervening regulatory boards.
