# Supplementary material for: Cortical morphology in patients with the deficit and non-deficit syndrome of schizophrenia: a worldwide meta- and mega-analyses
Source: Mol Psychiatry. 2023 Aug 29;28(10):4363–73. doi: 10.1038/s41380-023-02221-w (PMC10827665; doi:10.1038/s41380-023-02221-w)
Supplement: Supplementary file 1 — Supplementary information [file 41380_2023_2221_MOESM1_ESM.docx]

**Supplementary information**

**Supplementary Table S1.** Instruments for clinical assessment and inclusion/exclusion criteria

**Supplementary Table S2.** Demographical information for the Meta-Analysis sample

**Supplementary Table S3.** Demographical information for the Mega-Analysis sample

**Supplementary Table S4.** Clinical information for the Meta-Analysis sample

**Supplementary Table S5.** Clinical information for the Mega-Analysis sample

**Supplementary Table S6.** Sample image acquisition and image processing details

**Supplementary Table S7**. Results from random effect Meta-Analysis of left cortical thickness

**Supplementary Table S8**. Results from random effect Meta-Analysis of right cortical thickness

**Supplementary Table S9**. Results from random effect Meta-Analysis of left cortical surface area

**Supplementary Table S10**. Results from random effect Meta-Analysis of right cortical surface area

**Supplementary Table S11**. Regions excluded for significant heterogeneity in DSZ

**Supplementary Table S12**. Regions excluded for significant heterogeneity in NDSZ

**Supplementary Table S1.** Instruments for clinical assessment and inclusion/exclusion criteria.

| **Dataset** | **Country** | **Instruments for clinical**  **assessment** | **Sample characteristics/inclusion criteria** | **Exclusion criteria** |
| --- | --- | --- | --- | --- |
| **NPL_FSL** | Italy | Diagnosis was confirmed according to the DSM-IV using SCID-I | Inclusion criteria were (i) age between 18 and 65 years; (ii) at least five years of education; and (iii) suitability for MRI scanning. | (i) history of alcohol or drug abuse in the two years before the assessment; (ii) lifetime drug dependence; (iii) traumatic head injury with loss of consciousness; (iv) past or present major medical illness or neurological disorders; (v) any (for healthy controls) or additional (for patients) psychiatric disorder or mental retardation; (vi) dementia or cognitive deterioration according to DSM-IV-TR criteria, and MMSE score < 25, consistent with normative data in the Italian population; (vii) not able or not willing to give written informed consent. |
| **CAMH** | Canada | SCID DSM-IV-TR Axis I | Schizophrenia outpatients who were clinically stable as determined by no medication change within the past month. | (i) intelligence quotient < 70 as estimated by the WTAR; (ii) substance dependence or abuse reported or indicated by a urine toxicology screen; (iii) head trauma with loss of consciousness, neurological disorders; (iv) any magnetic resonance imaging contraindications; (v) a first degree relative with a primary psychotic disorder was also an exclusion criterion for controls. |
| **CIAM_UCT** | South Africa | DSM-IV using SCID-I | All recruited participants were aged between 19-40 years. Patients were currently stable outpatients with SCID-I confirmed DSM-IV diagnoses of schizophrenia. | Participants were excluded if they reported: (i) head injury; (ii) self /family history of epilepsy; or (iii) were receiving pharmaceutical treatment for a general medical condition. Healthy controls: (iv) any psychiatric disorder as ascertained the SCID-I. |
| **CLiNG** | Germany | ICD-10 and DSM-IV criteria, based on clinical interview and review of clinical history | Age between 18 and 60 years. Patients met the diagnostic criteria for schizophrenic disorders,  according to ICD-10 and DSM-IV classification standards. Controls were recruited from the local population. | For both controls and patients: (i) lifetime diagnoses of substance dependence, substance abuse during the last month, cannabis abuse during the last 2 weeks; (ii) mental retardation, dementia and neurological illnesses. Additional exclusion criteria for controls only: (iii) current or past psychiatric disorders; (iv) family history of psychiatric disorders. |
| **EDINBURG** | United Kingdom | SCID for DSM-IV | DSM-IV schizophrenia. | (i) alcohol misuse; (ii) head injury |
| **FBIRN** | United States | SCID for DSM-IV-TR Axis I Disorders (SCID-I/P & SCID-I/NP 11/2002 revision) | Individuals with schizophrenia who were clinically stable outpatients whose antipsychotic medications and doses had not changed within the last two months. Health subjects had similar mean age, sex, handedness, and race distributions as the schizophrenia group. | For both schizophrenia and healthy subjects: (i) history of major medical illness; (ii) drug dependence in the last 5 years (except for nicotine); (iii) current substance abuse disorder; or (iv) MRI contraindications. For schizophrenia patients only: (v) significant tardive dyskinesia. For healthy subjects: (vi) current or past history of major neurological or psychiatric illness; (viii) a first-degree relative with an Axis-I psychotic disorder diagnosis. |
| **FIDMAG** | Spain | Patients met DSM-IV criteria for schizophrenia,  based on interview and review of clinical history | All participants were in the 18-65 age range. | For healthy controls: (i) history of mental illness and/or treatment with  psychotropic medication. For patients: (i) history of brain trauma or  neurological disease or (ii) alcohol/  substance abuse within 12 months before participation. |
| **HMS** | Germany | ICD-10 and DSM-IV criteria, based on clinical interview and review of clinical history | Age between 18 and 60 years. Patients met the diagnostic criteria for schizophrenic disorders,  according to ICD-10 and DSM-IV classification standards. Healthy controls were recruited from the local population. | For both controls and patients: (i) lifetime diagnoses of substance dependence, substance abuse during the last month, cannabis abuse during the last 2 weeks; (ii) mental retardation, dementia and neurological illnesses. For controls only: (iii) current or past psychiatric disorders, family history of psychiatric disorders. |
| **LONDON** | United Kingdom | Diagnosis was made using Operational Criteria system according to ICD-10 criteria, using patient clinical notes for the month after their first contact with psychiatric services. | Patients aged 18-65 presenting to the South London and Maudsley National Health Service Foundation Trust, South East London, England, with a functional psychotic illness based on ICD-10 codes F10-19, excluding coding F1x.0 for acute intoxication; F20-29 and F30-39, psychosis codes were invited to participate. | (i) history of head trauma or injury with loss of consciousness lasting longer than 1 hour; (ii) history of any serious medical or surgical illness; (iii) learning disabilities; (iv) current or past organic psychosis; (v) lack of English fluency; (vi) known contraindications to conventional MRI. |

**Legend:** *DSM: Diagnostic and Statistical Manual of Mental Disorders; ICD: International Statistical Classification of Diseases; MMSE: Mini-Mental State Examination; MRI: Magnetic Resonance Imaging; SCID: Structured Clinical Interview for DSM; WTAR: Wechsler Test for Adult Reading.*

**Datasets:** CAMH: Center for Addiction and Mental Health; CIAM-UCT: Cortical Inhibition and Attentional Modulation: a study of psychosis - University of Cape Town; CLiNG: Clinical Neuroscience Göttingen; EDINBURG: University of Edinburgh; FBIRN: Function Biomedical Informatics Research Network; FIDMAG: Sisters Hospitallers Research Foundation; HMS: Homburg Multidiagnosis Study-Heidelberg University; LONDON: King's College London; NPL_FSL: Neuropsychiatry Laboratory, Fondazione Santa Lucia.

**Supplementary Table S2. Demographical information for the Meta-Analysis sample**

| **Dataset** | **N (TOT)** | **N (DSZ)** | **N (NDSZ)** | **N (HC)** | **M/F (DSZ)** | **M/F (NDSZ)** | **M/F (HC)** | **Age (DSZ) mean (sd)** | **Age (NDSZ) mean (sd)** | **Age (HC) mean (sd)** |
| --- | --- | --- | --- | --- | --- | --- | --- | --- | --- | --- |
| CAMH | 224 | 32 | 46 | 146 | 25/7 | 25/21 | 77/69 | 45.5 (17.07) | 41.87 (16.42) | 43.6 (19.06) |
| CIAM-UCT | 34 | 6 | 3 | 25 | 5/1 | 2/1 | 12/13 | 30.67 (7.48) | 33 (11.27) | 25.88 (4.39) |
| CLiNG | 365 | 10 | 32 | 323 | 9/1 | 22/10 | 132/191 | 32.5 (8.96) | 33.38 (9.71) | 25.18 (6.41) |
| EDINBURGH | 58 | 10 | 13 | 35 | 6/4 | 8/5 | 19/16 | 38.5 (12.58) | 38.23 (8.29) | 37.29 (15.21) |
| FBIRN | 333 | 45 | 114 | 174 | 40/5 | 83/31 | 124/50 | 37.52 (11.26) | 39.77 (11.58) | 35.8 (11.4) |
| FIDMAG | 193 | 41 | 29 | 123 | 32/9 | 21/8 | 69/54 | 39.27 (11.23) | 37.66 (11.44) | 37.54 (10.13) |
| HMS | 93 | 3 | 35 | 55 | 2/1 | 24/11 | 28/27 | 25.33 (6.81) | 28.94 (7.17) | 35.38 (10.02) |
| LONDON | 75 | 14 | 35 | 26 | 12/2 | 18/17 | 14/12 | 25.43 (5.65) | 28.43 (7.63) | 26.92 (7.82) |
| NPL_FSL | 185 | 7 | 66 | 112 | 7/1 | 41/25 | 71/41 | 36.29 (12.72) | 40.38 (11.88) | 37.49 (11.54) |

**Legend:** *DSZ, Deficit Schizophrenia; NDSZ Non Deficit Schizophrenia; HC, Healthy Controls; sd, standard deviation.*

**Datasets:** CAMH: Center for Addiction and Mental Health; CIAM-UCT: Cortical Inhibition and Attentional Modulation: a study of psychosis - University of Cape Town; CLiNG: Clinical Neuroscience Göttingen; EDINBURG: University of Edinburgh; FBIRN: Function Biomedical Informatics Research Network; FIDMAG: Sisters Hospitallers Research Foundation; HMS: Homburg Multidiagnosis Study-Heidelberg University; LONDON: King's College London; NPL_FSL: Neuropsychiatry Laboratory, Fondazione Santa Lucia.

**Supplementary Table S3. Demographical information for the Mega-Analysis sample**

| **Dataset** | **N (TOT)** | **N (DSZ)** | **N (NDSZ)** | **N (HC)** | **M/F (DSZ)** | **M/F (NDSZ)** | **M/F (HC)** | **Age (DSZ) mean (sd)** | **Age (NDSZ) mean (sd)** | **Age (HC) mean (sd)** |
| --- | --- | --- | --- | --- | --- | --- | --- | --- | --- | --- |
| CAMH | 161 | 26 | 43 | 92 | 22/4 | 23/20 | 45/47 | 42.04 (15.83) | 40.71 (15.65) | 44.89 (16.07) |
| CIAM-UCT | 26 | 6 | 3 | 17 | 5/1 | 2/1 | 7/10 | 32.8 (5.93) | 29.5 (13.44) | 28.06 (3.44) |
| CLiNG | 266 | 10 | 32 | 224 | 9/1 | 22/10 | 87/137 | 32.5 (8.96) | 33.38 (9.71) | 26.61 (5.73) |
| EDINBURGH | 55 | 10 | 13 | 32 | 6/4 | 8/5 | 15/17 | 38.5 (12.58) | 37 (7.31) | 39.28 (14.62) |
| FIDMAG | 120 | 40 | 28 | 52 | 31/9 | 21/7 | 22/30 | 38.33 (10.53) | 36.44 (10.25) | 38.48 (10.39) |
| HMS | 88 | 3 | 34 | 51 | 2/1 | 23/11 | 26/25 | 25.33 (6.81) | 29.39 (7.11) | 36.63 (9.29) |
| LONDON | 68 | 14 | 34 | 20 | 12/2 | 18/16 | 9/11 | 28 (8.25) | 29.17 (7.6) | 28.6 (8.2) |
| NPL_FSL | 160 | 6 | 67 | 87 | 6/0 | 43/24 | 61/26 | 40.83 (8.91) | 40 (11.18) | 40.95 (10.66) |

**Legend:** *DSZ, Deficit Schizophrenia; NDSZ Non Deficit Schizophrenia; HC, Healthy Controls; sd, standard deviation.*

**Datasets:** CAMH: Center for Addiction and Mental Health; CIAM-UCT: Cortical Inhibition and Attentional Modulation: a study of psychosis - University of Cape Town; CLiNG: Clinical Neuroscience Göttingen; EDINBURG: University of Edinburgh; FIDMAG: Sisters Hospitallers Research Foundation; HMS: Homburg Multidiagnosis Study-Heidelberg University; LONDON: King's College London; NPL_FSL: Neuropsychiatry Laboratory, Fondazione Santa Lucia.

**Supplementary Table S4. Clinical information for the Meta-Analysis sample**

| **Dataset** | **PANSS Total (DSZ) mean (sd)** | **PANSS Total (NDSZ) mean (sd)** | **CPZ (DSZ) mean (sd)** | **CPZ (NDSZ) mean (sd)** | **Medication A/T/B/N (DSZ)** | **Medication A/T/B/N (NDSZ)** |
| --- | --- | --- | --- | --- | --- | --- |
| CAMH | 55.31 (16.72) | 54 (15.63) | 238.63 (216.79) | 341.90 (347.66) | 23/4/1/4 | 32/2/3/9 |
| CIAM-UCT | 50.83 (16.22) | 54.33 (19.86) | 300 (357.77) | 50 (70.71) | 2/2/1/1 | 0/2/0/1 |
| CLiNG | 46.9 (11.07) | 53.63 (11.8) | 516.67 (514.3) | 692.97 (519.77) | 7/0/1/2 | 25/0/2/5 |
| EDINBURGH | 55.8 (18.9) | 52.31 (19.01) | 592.67 (450.51) | 242.29 (216.66) | 9/0/0/1 | 10/0/0/3 |
| FBIRN | 62.17 (16.66) | 58.59 (13.7) | 335 (229) | 379 (400) | 34/7/1/3 | 86/11/8/9 |
| FIDMAG | 72.93 (16.84) | 78.1 (19.67) | 501.49 (506.63) | 501.23 (258.06) | NA | NA |
| HMS | 90.33 (25.58) | 92.77 (16.7) | 305.56 (191.73) | 342.65 (360.51) | 3/0/0/0 | 31/0/0/4 |
| LONDON | 62.93 (11.52) | 58.29 (13.89) | 89.2 (9.89) | 95.54 (8.82) | NA | NA |
| NPL_FSL | 96.86 (16.12) | 90.02 (19.44) | 300 (176.1) | 442.45 (603.13) | 4/0/3/0 | 35/13/13/5 |

**Legend:** *DSZ, Deficit Schizophrenia; NDSZ, Non Deficit Schizophrenia; HC, Healthy Controls; sd, standard deviation.*

**Datasets:** CAMH: Center for Addiction and Mental Health; CIAM-UCT: Cortical Inhibition and Attentional Modulation: a study of psychosis - University of Cape Town; CLiNG: Clinical Neuroscience Göttingen; EDINBURG: University of Edinburgh; FBIRN: Function Biomedical Informatics Research Network; FIDMAG: Sisters Hospitallers Research Foundation; HMS: Homburg Multidiagnosis Study-Heidelberg University; LONDON: King's College London; NPL_FSL: Neuropsychiatry Laboratory, Fondazione Santa Lucia.

**Supplementary Table S5. Clinical information for the Mega-Analysis sample**

| **Dataset** | **PANSS Total (DSZ) mean (sd)** | **PANSS Total (NDSZ) mean (sd)** | **CPZ (DSZ) mean (sd)** | **CPZ (NDSZ) mean (sd)** | **Medication A/T/B/N (DSZ)** | **Medication A/T/B/N (NDSZ)** |
| --- | --- | --- | --- | --- | --- | --- |
| CAMH | 52.5 (15.74) | 55.2 (15.81) | 254.89 (229.39) | 349.84 (357.51) | 19/3/1/3 | 29/2/3/9 |
| CIAM-UCT | 54.4 (15.27) | 43 (4.24) | 300 (400) | 50 (70.71) | 2/2/1/1 | 0/2/0/1 |
| CLiNG | 46.9 (11.07) | 53.63 (11.8) | 516.67 (514.3) | 692.97 (519.77) | 7/0/1/2 | 25/0/2/5 |
| EDINBURGH | 55.8 (18.9) | 52.08 (19.84) | 592.67 (450.51) | 242.29 (216.66) | 9/0/0/1 | 10/0/0/3 |
| FIDMAG | 71.67 (17.37) | 76.88 (20.63) | 504.92 (513.38) | 500.6 (263.36) | NA | NA |
| HMS | 90.33 (25.58) | 92.12 (16.49) | 305.56 (191.73) | 320.2 (341.13) | 3/0/0/0 | 30/0/0/4 |
| LONDON | 60.8 (5.89) | 54.96 (12.5) | 89.2 (9.89) | 95.64 (9.01) | NA | NA |
| NPL_FSL | 97.17 (17.63) | 89.45 (19.49) | 316.67 (186.76) | 451.82 (595.62 | 3/0/3/0 | 35/15/13/4 |

**Legend:** *A, Atypical; B, Both; DSZ, Deficit Schizophrenia; HC, Healthy Controls; N, None; NDSZ, Non Deficit Schizophrenia; sd, standard deviation; T, Typical.*

**Datasets:** CAMH: Center for Addiction and Mental Health; CIAM-UCT: Cortical Inhibition and Attentional Modulation: a study of psychosis - University of Cape Town; CLiNG: Clinical Neuroscience Göttingen; EDINBURG: University of Edinburgh; FIDMAG: Sisters Hospitallers Research Foundation; HMS: Homburg Multidiagnosis Study-Heidelberg University; LONDON: King's College London; NPL_FSL: Neuropsychiatry Laboratory, Fondazione Santa Lucia.

**Supplementary Table S6. Sample image acquisition and image processing details**

| **Dataset** | **Number of scanners** | **Scanner Vendor & Type** | **Imaging Protocols** | **Slice orientation** | **FreeSurfer Version** | **Operating System** | **Excluded subjects for QC** |
| --- | --- | --- | --- | --- | --- | --- | --- |
| NPL_FSL | 1 | Siemens 3T Allegra | 3D MPRAGE: TE/TR = 2.4/7.92 ms, flip angle=15°, voxel size 1×1×1 mm | Sagittal | 6.0dev | Mac OSX | 3 |
| CAMH | 1 | GE 1.5T | SPGR, TR/TE/TI=12.3/5.3/300ms, flip angle=20°, 256x256x128 matrix, FOV=240x240mm, slice thickness=1.5mm | Axial | v5.3.0 | xubuntu x86_64- linux | 0 |
| CIAM-UCT | 1 | Siemens 3T Allegra | 3DMPRGE, 160 slices, TR=2300 ms, TE=3.93 ms, TI=1100 ms, resolution 1.3×1×1 mm3, 256 mm FOV | Sagittal | 5.3 stable | Linux | NA |
| CLiNG | 1 | 3T Magnetom TIM Trio | MRI scanning was performed on a 3.0- Tesla Magnetom TIM Trio (Siemens, Erlangen, Germany). A T1-weighted, 3D magnetization prepared rapid gradient echo sequence (MPRAGE) (TR/TE/TI/FA=2250 ms/3.26 ms/900 ms/9°; image matrix = 256 x 256; duration 8 min and 26 sec) was acquired generating 192 sagittal slices with a voxel size of 1 mm3.” | Sagittal | v5.3.0 | Ubuntu 12.04 | 0 |
| EDINBURG | 1 | 3T Siemans Verio | Used T1-weighted, magnetisation prepared rapid acquisition gradient echo (MP-RAGE) sequence prescribed using the AC-PC line, providing 160 saggital slices of 1.0mm thickness, with 256 x 256mm2 field of view, matrix size 256 x 256mm2. Further scan parameters – repetition time = 2300ms, echo time = 2.98ms, inversion time = 900ms and flip angle = 9degress. | Sagittal | v5.0.1 | Linux | 0 |
| FBIRN | 7 | 6 x 3T Siemens Tim Trio; 1 x 3T GE Discovery MR750 | High-resolution structural imaging scans were acquired on six 3T Siemens Tim® Trio System and one 3T General Electric Discovery MR750 scanner. MP-RAGE scan parameters for the Siemens scanner were: scan plane=sagittal, TR/TE/TI=2300/2.94/1100ms, GRAPPA acceleration factor=2, flip angle=9°, resolution=256×256x160, FOV=220mm2, voxel size=0.86x0.86x1.2mm, and NEX=1. IR-SPGR scan parameters for the General Electric scanner were: scan plane=sagittal, TR/TE/TI=5.95/1.99/450ms, ASSET acceleration factor=2, a flip angle=12°, resolution=256×256x166, FOV=220mm2, voxel size=0.86x0.86x1.2mm, and NEX=1. All scans covered the entire brain. | Sagittal | 5.3 stable | Linux - Centos 64bit | 0 |
| FIDMAG | 1 | GE 1.5T Signa | T1w: TE/TR/TI=3.93/2000/710ms,flip angle=15 degrees, voxel size=0.94x1.07x1mm interpolated to voxel size=0.47x0.47x1mm | Axial | 5.3dev | Ubuntu | NA |
| HMS | 1 | 1.5 T Magnetom Sonata | MRI scanning was performed on a 1.5- Tesla Magnetom Sonata (Siemens, Erlangen, Germany). A T1-weighted, magnetization prepared rapid gradient echo sequence (MPRAGE) (TR/TE/TI/FA=1900 ms/4.0 ms/700 ms/15°; image matrix = 256 x 256) was acquired generating 176 consecutive sagittal slices with a voxel size of 1 mm3. ~5 min | Sagittal | v 5.1.0 | centos6 x86_64 | 0 |
| LONDON | NA | NA | NA | NA | NA | NA | NA |

**Datasets:** CAMH: Center for Addiction and Mental Health; CIAM-UCT: Cortical Inhibition and Attentional Modulation: a study of psychosis - University of Cape Town; CLiNG: Clinical Neuroscience Göttingen; EDINBURG: University of Edinburgh; FBIRN: Function Biomedical Informatics Research Network; FIDMAG: Sisters Hospitallers Research Foundation; HMS: Homburg Multidiagnosis Study-Heidelberg University; LONDON: King's College London; NPL_FSL: Neuropsychiatry Laboratory, Fondazione Santa Lucia.

**Supplementary Table S7**. Results from random effect Meta-Analysis of left cortical thickness

| **Cortical**  **Regions** | **Groups** | **Effect size and 95% confidence interval** | | | | | | **Test of null**  **(2-Tail)** | | **Heterogeneity** | | | | **Sub-groups Analysis** | | | | |
| --- | --- | --- | --- | --- | --- | --- | --- | --- | --- | --- | --- | --- | --- | --- | --- | --- | --- | --- |
|  |  | **N** | **Point estim** | **Std err** | **Var** | **Low limit** | **Up limit** | **Z-value** | ***P-***  ***value*** | **Q-value** | **df** | ***P-value*** | **I-**  **sqrd** | **Tau- Sqrd** |  | **Q-value** | **df** | ***P-value*** |
| **Banks sts** | DSZ | 9 | -0.46 | 0.11 | 0.01 | -0.68 | -0.24 | -4.08 | <0.0001 | 11.17 | 8 | 0.1924 | 28.36 | 0.03 | Total between | 0.829 | 1 | 0.362 |
|  | NDSZ | 9 | -0.32 | 0.10 | 0.01 | -0.52 | -0.12 | -3.19 | 0.0014 | 16.91 | 8 | 0.0310 | 52.70 | 0.04 |  |  |  |  |
| **Caudal**  **anterior**  **cingulate** | DSZ | 9 | -0.03 | 0.09 | 0.01 | -0.20 | 0.14 | -0.36 | 0.7158 | 7.86 | 8 | 0.4470 | 0.00 | 0.00 | Total between | 1.111 | 1 | 0.292 |
|  | NDSZ | 9 | -0.16 | 0.09 | 0.01 | -0.35 | 0.02 | -1.77 | 0.0769 | 14.98 | 8 | 0.0596 | 46.59 | 0.03 |  |  |  |  |
| **Caudal**  **middle**  **frontal** | DSZ | 9 | -0.59 | 0.11 | 0.01 | -0.80 | -0.37 | -5.37 | <0.0001 | 11.12 | 8 | 0.1948 | 28.08 | 0.03 | Total between | 1.944 | 1 | 0.163 |
|  | NDSZ | 9 | -0.41 | 0.06 | 0.00 | -0.54 | -0.29 | -6.46 | <0.0001 | 6.68 | 8 | 0.5712 | 0.00 | 0.00 |  |  |  |  |
| **Cuneus** | DSZ | 9 | -0.23 | 0.09 | 0.01 | -0.40 | -0.06 | -2.72 | 0.0066 | 6.92 | 8 | 0.5453 | 0.00 | 0.00 | Total between | 0.154 | 1 | 0.694 |
|  | NDSZ | 9 | -0.18 | 0.09 | 0.01 | -0.36 | 0.00 | -1.99 | 0.0465 | 14.62 | 8 | 0.0671 | 45.26 | 0.03 |  |  |  |  |
| **Entorhinal** | DSZ | 9 | -0.12 | 0.16 | 0.03 | -0.44 | 0.21 | -0.71 | 0.4807 | 24.19 | 8 | 0.0021 | 66.92 | 0.15 | Total between | 0.064 | 1 | 0.801 |
|  | NDSZ | 9 | -0.17 | 0.13 | 0.02 | -0.43 | 0.09 | -1.28 | 0.2014 | 29.90 | 8 | 0.0002 | 73.25 | 0.11 |  |  |  |  |
| **Frontal pole** | DSZ | 9 | -0.38 | 0.12 | 0.01 | -0.62 | -0.15 | -3.22 | 0.0013 | 12.83 | 8 | 0.1178 | 37.65 | 0.04 | Total between | 0.732 | 1 | 0.392 |
|  | NDSZ | 9 | -0.27 | 0.07 | 0.00 | -0.40 | -0.14 | -3.98 | <0.0001 | 8.61 | 8 | 0.3764 | 7.06 | 0.00 |  |  |  |  |
| **Fusiform** | DSZ | 9 | -0.43 | 0.18 | 0.03 | -0.77 | -0.08 | -2.41 | 0.0158 | 27.64 | 8 | 0.0005 | 71.05 | 0.18 | Total between | 0.001 | 1 | 0.976 |
|  | NDSZ | 9 | -0.43 | 0.17 | 0.03 | -0.77 | -0.09 | -2.51 | 0.0122 | 50.62 | 8 | <0.0001 | 84.20 | 0.21 |  |  |  |  |
| **Inferior**  **parietal** | DSZ | 9 | -0.48 | 0.09 | 0.01 | -0.67 | -0.30 | -5.14 | <0.0001 | 8.89 | 8 | 0.3516 | 10.02 | 0.01 | Total between | 1.244 | 1 | 0.265 |
|  | NDSZ | 9 | -0.35 | 0.08 | 0.01 | -0.50 | -0.19 | -4.42 | <0.0001 | 10.96 | 8 | 0.2040 | 27.00 | 0.01 |  |  |  |  |
| **Inferior**  **temporal** | DSZ | 9 | -0.35 | 0.18 | 0.03 | -0.70 | -0.01 | -2.00 | 0.0458 | 27.14 | 8 | 0.0007 | 70.52 | 0.18 | Total between | 0.068 | 1 | 0.795 |
|  | NDSZ | 9 | -0.29 | 0.15 | 0.02 | -0.58 | 0.00 | -1.98 | 0.0475 | 36.55 | 8 | <0.0001 | 78.11 | 0.14 |  |  |  |  |
| **Insula** | DSZ | 9 | -0.38 | 0.16 | 0.03 | -0.69 | -0.06 | -2.34 | 0.0195 | 22.82 | 8 | 0.0036 | 64.95 | 0.14 | Total between | 0.032 | 1 | 0.859 |
|  | NDSZ | 9 | -0.34 | 0.15 | 0.02 | -0.64 | -0.04 | -2.19 | 0.0284 | 39.92 | 8 | <0.0001 | 79.96 | 0.16 |  |  |  |  |
| **Isthmus**  **cingulate** | DSZ | 9 | -0.39 | 0.12 | 0.01 | -0.62 | -0.16 | -3.30 | 0.0010 | 12.79 | 8 | 0.1192 | 37.46 | 0.04 | Total between | 0.503 | 1 | 0.478 |
|  | NDSZ | 9 | -0.28 | 0.10 | 0.01 | -0.47 | -0.09 | -2.88 | 0.0040 | 16.45 | 8 | 0.0364 | 51.36 | 0.04 |  |  |  |  |
| **Lateral**  **occipital** | DSZ | 9 | -0.31 | 0.17 | 0.03 | -0.64 | 0.03 | -1.81 | 0.0706 | 26.03 | 8 | 0.0010 | 69.27 | 0.16 | Total between | 0.010 | 1 | 0.922 |
|  | NDSZ | 9 | -0.33 | 0.15 | 0.02 | -0.62 | -0.04 | -2.25 | 0.0244 | 35.97 | 8 | <0.0001 | 77.76 | 0.14 |  |  |  |  |
| **Lateral**  **orbitofrontal** | DSZ | 9 | -0.40 | 0.18 | 0.03 | -0.75 | -0.04 | -2.20 | 0.0279 | 28.64 | 8 | 0.0004 | 72.06 | 0.19 | Total between | 0.148 | 1 | 0.700 |
|  | NDSZ | 9 | -0.30 | 0.16 | 0.02 | -0.61 | 0.00 | -1.94 | 0.0527 | 41.72 | 8 | <0.0001 | 80.83 | 0.16 |  |  |  |  |
| **Lingual** | DSZ | 9 | -0.32 | 0.17 | 0.03 | -0.66 | 0.02 | -1.85 | 0.0640 | 26.29 | 8 | 0.0009 | 69.57 | 0.17 | Total between | 0.021 | 1 | 0.886 |
|  | NDSZ | 9 | -0.29 | 0.14 | 0.02 | -0.56 | -0.01 | -2.05 | 0.0408 | 33.21 | 8 | <0.0001 | 75.91 | 0.12 |  |  |  |  |
| **Medial orbito-frontal** | DSZ | 9 | -0.12 | 0.17 | 0.03 | -0.44 | 0.21 | -0.70 | 0.4813 | 23.76 | 8 | 0.0025 | 66.34 | 0.14 | Total between | 0.007 | 1 | 0.935 |
|  | NDSZ | 9 | -0.10 | 0.13 | 0.02 | -0.35 | 0.15 | -0.77 | 0.4391 | 28.09 | 8 | 0.0005 | 71.52 | 0.10 |  |  |  |  |
| **Middle**  **temporal** | DSZ | 9 | -0.51 | 0.17 | 0.03 | -0.84 | -0.18 | -3.06 | 0.0022 | 23.46 | 8 | 0.0028 | 65.90 | 0.15 | Total between | 0.743 | 1 | 0.389 |
|  | NDSZ | 9 | -0.32 | 0.14 | 0.02 | -0.60 | -0.04 | -2.25 | 0.0246 | 33.85 | 8 | <0.0001 | 76.36 | 0.13 |  |  |  |  |
| **Paracentral** | DSZ | 9 | -0.35 | 0.12 | 0.01 | -0.58 | -0.12 | -3.01 | 0.0026 | 12.30 | 8 | 0.1383 | 34.96 | 0.04 | Total between | 1.157 | 1 | 0.282 |
|  | NDSZ | 9 | -0.19 | 0.09 | 0.01 | -0.36 | -0.02 | -2.18 | 0.0291 | 13.43 | 8 | 0.0980 | 40.42 | 0.03 |  |  |  |  |
| **Para-hippocampal** | DSZ | 9 | -0.32 | 0.10 | 0.01 | -0.52 | -0.13 | -3.28 | 0.0010 | 9.54 | 8 | 0.2992 | 16.10 | 0.01 | Total between | 0.118 | 1 | 0.731 |
|  | NDSZ | 9 | -0.28 | 0.10 | 0.01 | -0.47 | -0.08 | -2.82 | 0.0048 | 16.38 | 8 | 0.0372 | 51.17 | 0.04 |  |  |  |  |
| **Pars**  **opercularis** | DSZ | 9 | -0.57 | 0.13 | 0.02 | -0.83 | -0.31 | -4.30 | <0.0001 | 15.51 | 8 | 0.0500 | 48.40 | 0.07 | Total between | 1.428 | 1 | 0.232 |
|  | NDSZ | 9 | -0.39 | 0.07 | 0.00 | -0.52 | -0.26 | -5.72 | <0.0001 | 8.80 | 8 | 0.3595 | 9.09 | 0.00 |  |  |  |  |
| **Pars**  **orbitalis** | DSZ | 9 | -0.15 | 0.18 | 0.03 | -0.51 | 0.20 | -0.83 | 0.4049 | 29.62 | 8 | 0.0002 | 72.99 | 0.19 | Total between | 0.004 | 1 | 0.947 |
|  | NDSZ | 9 | -0.17 | 0.12 | 0.01 | -0.40 | 0.07 | -1.36 | 0.1747 | 25.37 | 8 | 0.0013 | 68.46 | 0.08 |  |  |  |  |
| **Pars**  **triangularis** | DSZ | 9 | -0.32 | 0.11 | 0.01 | -0.55 | -0.10 | -2.88 | 0.0040 | 11.80 | 8 | 0.1603 | 32.21 | 0.03 | Total between | 0.015 | 1 | 0.901 |
|  | NDSZ | 9 | -0.31 | 0.11 | 0.01 | -0.52 | -0.09 | -2.73 | 0.0063 | 21.08 | 8 | 0.0069 | 62.05 | 0.06 |  |  |  |  |
|  | DSZ | 9 | -0.07 | 0.09 | 0.01 | -0.24 | 0.10 | -0.82 | 0.4107 | 3.61 | 8 | 0.8908 | 0.00 | 0.00 | Total between | 0.541 | 1 | 0.462 |
| **Pericalcarine** | NDSZ | 9 | 0.01 | 0.06 | 0.00 | -0.12 | 0.13 | 0.13 | 0.9005 | 5.48 | 8 | 0.7056 | 0.00 | 0.00 |  |  |  |  |
| **Postcentral** | DSZ | 9 | -0.49 | 0.12 | 0.01 | -0.73 | -0.25 | -4.03 | <0.0001 | 13.37 | 8 | 0.0999 | 40.14 | 0.05 | Total between | 0.978 | 1 | 0.323 |
|  | NDSZ | 9 | -0.34 | 0.09 | 0.01 | -0.52 | -0.16 | -3.63 | 0.0003 | 14.91 | 8 | 0.0609 | 46.35 | 0.03 |  |  |  |  |
| **Posterior**  **cingulate** | DSZ | 9 | -0.34 | 0.11 | 0.01 | -0.55 | -0.13 | -3.20 | 0.0014 | 10.81 | 8 | 0.2129 | 25.98 | 0.03 | Total between | 0.000 | 1 | 0.998 |
|  | NDSZ | 9 | -0.34 | 0.10 | 0.01 | -0.55 | -0.14 | -3.29 | 0.0010 | 18.33 | 8 | 0.0189 | 56.37 | 0.05 |  |  |  |  |
| **Precentral** | DSZ | 9 | -0.54 | 0.13 | 0.02 | -0.80 | -0.29 | -4.19 | <0.0001 | 15.05 | 8 | 0.0582 | 46.85 | 0.06 | Total between | 1.945 | 1 | 0.163 |
|  | NDSZ | 9 | -0.33 | 0.09 | 0.01 | -0.50 | -0.15 | -3.71 | 0.0002 | 13.37 | 8 | 0.0997 | 40.18 | 0.03 |  |  |  |  |
| **Precuneus** | DSZ | 9 | -0.37 | 0.10 | 0.01 | -0.57 | -0.16 | -3.49 | 0.0005 | 10.43 | 8 | 0.2364 | 23.26 | 0.02 | Total between | 0.440 | 1 | 0.507 |
|  | NDSZ | 9 | -0.26 | 0.12 | 0.01 | -0.49 | -0.02 | -2.17 | 0.0301 | 24.18 | 8 | 0.0021 | 66.92 | 0.08 |  |  |  |  |
| **Rostral**  **anterior**  **cingulate** | DSZ | 9 | -0.35 | 0.18 | 0.03 | -0.70 | 0.00 | -1.97 | 0.0486 | 28.01 | 8 | 0.0005 | 71.43 | 0.18 | Total between | 0.357 | 1 | 0.550 |
|  | NDSZ | 9 | -0.21 | 0.15 | 0.02 | -0.50 | 0.07 | -1.47 | 0.1409 | 36.22 | 8 | <0.0001 | 77.91 | 0.14 |  |  |  |  |
| **Rostral**  **middle**  **frontal** | DSZ | 9 | -0.51 | 0.17 | 0.03 | -0.85 | -0.17 | -2.98 | 0.0029 | 26.28 | 8 | 0.0009 | 69.56 | 0.17 | Total between | 0.381 | 1 | 0.537 |
|  | NDSZ | 9 | -0.38 | 0.12 | 0.01 | -0.62 | -0.14 | -3.13 | 0.0017 | 24.96 | 8 | 0.0016 | 67.95 | 0.08 |  |  |  |  |
| **Superior**  **frontal** | DSZ | 9 | -0.71 | 0.17 | 0.03 | -1.04 | -0.37 | -4.15 | <0.0001 | 25.65 | 8 | 0.0012 | 68.81 | 0.16 | Total between | 0.570 | 1 | 0.450 |
|  | NDSZ | 9 | -0.56 | 0.10 | 0.01 | -0.76 | -0.36 | -5.45 | <0.0001 | 17.59 | 8 | 0.0245 | 54.53 | 0.05 |  |  |  |  |
| **Superior**  **parietal** | DSZ | 9 | -0.33 | 0.09 | 0.01 | -0.50 | -0.16 | -3.87 | 0.0001 | 3.86 | 8 | 0.8693 | 0.00 | 0.00 | Total between | 1.063 | 1 | 0.303 |
|  | NDSZ | 9 | -0.22 | 0.07 | 0.00 | -0.35 | -0.08 | -3.15 | 0.0017 | 9.06 | 8 | 0.3373 | 11.70 | 0.01 |  |  |  |  |
| **Superior**  **temporal** | DSZ | 9 | -0.54 | 0.16 | 0.02 | -0.85 | -0.23 | -3.40 | 0.0007 | 20.73 | 8 | 0.0079 | 61.41 | 0.12 | Total between | 0.406 | 1 | 0.524 |
|  | NDSZ | 9 | -0.41 | 0.12 | 0.01 | -0.64 | -0.18 | -3.52 | 0.0004 | 21.97 | 8 | 0.0050 | 63.58 | 0.07 |  |  |  |  |
| **Supramarginal** | DSZ | 9 | -0.57 | 0.09 | 0.01 | -0.74 | -0.40 | -6.54 | <0.0001 | 5.64 | 8 | 0.6870 | 0.00 | 0.00 | Total between | 3.497 | 1 | 0.061 |
|  | NDSZ | 9 | -0.37 | 0.06 | 0.00 | -0.49 | -0.24 | -5.77 | <0.0001 | 6.77 | 8 | 0.5617 | 0.00 | 0.00 |  |  |  |  |
| **Temporal pole** | DSZ | 9 | -0.27 | 0.13 | 0.02 | -0.52 | -0.02 | -2.14 | 0.0320 | 14.47 | 8 | 0.0702 | 44.73 | 0.06 | Total between | 0.051 | 1 | 0.822 |
|  | NDSZ | 9 | -0.24 | 0.10 | 0.01 | -0.43 | -0.04 | -2.38 | 0.0174 | 16.79 | 8 | 0.0324 | 52.36 | 0.04 |  |  |  |  |
| **Transverse**  **temporal** | DSZ | 9 | -0.33 | 0.11 | 0.01 | -0.54 | -0.13 | -3.15 | 0.0016 | 10.47 | 8 | 0.2337 | 23.58 | 0.02 | Total between | 0.806 | 1 | 0.369 |
|  | NDSZ | 9 | -0.22 | 0.08 | 0.01 | -0.36 | -0.07 | -2.86 | 0.0043 | 10.30 | 8 | 0.2448 | 22.30 | 0.01 |  |  |  |  |
| **Mean thickness** | DSZ | 9 | -0.67 | 0.15 | 0.02 | -0.97 | -0.37 | -4.40 | <0.0001 | 20.42 | 8 | 0.0088 | 60.83 | 0.11 | Total between | 1.901 | 1 | 0.168 |
|  | NDSZ | 9 | -0.50 | 0.13 | 0.02 | -0.76 | -0.24 | -3.74 | 0.0002 | 29.46 | 8 | 0.0003 | 72.84 | 0.11 |  |  |  |  |

**Legend:** *DSZ, Deficit Schizophrenia; NDSZ, Non Deficit Schizophrenia; Point estim, estimated standard difference; Std err, Standard Error; Var, Variance; df, degrees of freedom; Sqrd, Squared*

**Supplementary Table S8**. Results from random effect Meta-Analysis of right cortical thickness

| **Cortical**  **Regions** | **Groups** | **Effect size and 95% confidence interval** | | | | | | **Test of null**  **(2-Tail)** | | **Heterogeneity** | | | |  | **Sub-groups Analysis** | | | |
| --- | --- | --- | --- | --- | --- | --- | --- | --- | --- | --- | --- | --- | --- | --- | --- | --- | --- | --- |
|  |  | **N** | **Point estim** | **Std err** | **Var** | **Low limit** | **Up limit** | **Z-value** | ***P-***  ***value*** | **Q-value** | **df** | ***P-value*** | **I-**  **sqrd** | **Tau- Sqrd** |  | **Q-value** | **df** | **P-value** |
| **Bankssts** | DSZ | 9 | -0.53 | 0.11 | 0.01 | -0.75 | -0.32 | -4.82 | <0.0001 | 11.20 | 8 | 0.1907 | 28.56 | 0.02 | Total between | 1.251 | 1 | 0.263 |
|  | NDSZ | 9 | -0.38 | 0.08 | 0.01 | -0.54 | -0.21 | -4.50 | <0.0001 | 12.31 | 8 | 0.1379 | 35.01 | 0.02 |  |  |  |  |
| **Caudal**  **anterior**  **cingulate** | DSZ | 9 | -0.19 | 0.13 | 0.02 | -0.44 | 0.06 | -1.47 | 0.1424 | 15.09 | 8 | 0.0574 | 46.99 | 0.05 | Total between | 0.172 | 1 | 0.679 |
|  | NDSZ | 9 | -0.12 | 0.10 | 0.01 | -0.33 | 0.08 | -1.15 | 0.2486 | 18.78 | 8 | 0.0161 | 57.39 | 0.05 |  |  |  |  |
| **Caudal**  **middle**  **frontal** | DSZ | 9 | -0.47 | 0.09 | 0.01 | -0.64 | -0.31 | -5.51 | <0.0001 | 4.89 | 8 | 0.7688 | 0.00 | 0.02 | Total between | 1.699 | 1 | 0.192 |
|  | NDSZ | 9 | -0.32 | 0.08 | 0.01 | -0.48 | -0.16 | -3.91 | <0.0001 | 11.76 | 8 | 0.1621 | 32.00 | 0.02 |  |  |  |  |
| **Cuneus** | DSZ | 9 | -0.21 | 0.10 | 0.01 | -0.41 | -0.01 | -2.05 | 0.0408 | 9.99 | 8 | 0.2660 | 19.89 | 0.02 | Total between | 0.001 | 1 | 0.976 |
|  | NDSZ | 9 | -0.21 | 0.08 | 0.01 | -0.36 | -0.05 | -2.60 | 0.0094 | 11.13 | 8 | 0.1946 | 28.10 | 0.02 |  |  |  |  |
| **Entorhinal** | DSZ | 9 | -0.23 | 0.18 | 0.03 | -0.59 | 0.12 | -1.28 | 0.1993 | 29.79 | 8 | 0.0002 | 73.15 | 0.16 | Total between | 0.008 | 1 | 0.929 |
|  | NDSZ | 9 | -0.26 | 0.15 | 0.02 | -0.56 | 0.05 | -1.66 | 0.0972 | 40.44 | 8 | <0.0001 | 80.22 | 0.16 |  |  |  |  |
| **Frontal pole** | DSZ | 9 | -0.25 | 0.09 | 0.01 | -0.42 | -0.09 | -2.96 | 0.0031 | 5.64 | 8 | 0.6880 | 0.00 | 0.00 | Total between | 0.409 | 1 | 0.523 |
|  | NDSZ | 9 | -0.18 | 0.07 | 0.00 | -0.32 | -0.05 | -2.70 | 0.0069 | 8.83 | 8 | 0.3570 | 9.38 | 0.00 |  |  |  |  |
| **Fusiform** | DSZ | 9 | -0.54 | 0.16 | 0.03 | -0.85 | -0.23 | -3.38 | 0.0007 | 22.42 | 8 | 0.0042 | 64.32 | 0.12 | Total between | 0.359 | 1 | 0.549 |
|  | NDSZ | 9 | -0.41 | 0.14 | 0.02 | -0.69 | -0.14 | -2.94 | 0.0033 | 33.20 | 8 | <0.0001 | 75.91 | 0.12 |  |  |  |  |
| **Inferior**  **parietal** | DSZ | 9 | -0.49 | 0.11 | 0.01 | -0.71 | -0.27 | -4.34 | <0.0001 | 11.73 | 8 | 0.1637 | 31.79 | 0.05 | Total between | 0.468 | 1 | 0.494 |
|  | NDSZ | 9 | -0.38 | 0.11 | 0.01 | -0.59 | -0.18 | -3.63 | 0.0003 | 18.97 | 8 | 0.0150 | 57.82 | 0.05 |  |  |  |  |
| **Inferior**  **temporal** | DSZ | 9 | -0.44 | 0.14 | 0.02 | -0.72 | -0.16 | -3.10 | 0.0019 | 17.41 | 8 | 0.0261 | 54.05 | 0.13 | Total between | 0.100 | 1 | 0.752 |
|  | NDSZ | 9 | -0.38 | 0.14 | 0.02 | -0.66 | -0.09 | -2.62 | 0.0088 | 33.99 | 8 | <0.0001 | 76.46 | 0.13 |  |  |  |  |
| **Insula** | DSZ | 9 | -0.31 | 0.16 | 0.03 | -0.62 | 0.01 | -1.91 | 0.0557 | 23.00 | 8 | 0.0034 | 65.21 | 0.15 | Total between | 0.048 | 1 | 0.827 |
|  | NDSZ | 9 | -0.26 | 0.15 | 0.02 | -0.55 | 0.03 | -1.74 | 0.0827 | 38.27 | 8 | <0.0001 | 79.09 | 0.15 |  |  |  |  |
| **Isthmus**  **cingulate** | DSZ | 9 | -0.35 | 0.13 | 0.02 | -0.61 | -0.10 | -2.72 | 0.0066 | 15.10 | 8 | 0.0573 | 47.02 | 0.05 | Total between | 0.041 | 1 | 0.839 |
|  | NDSZ | 9 | -0.32 | 0.10 | 0.01 | -0.52 | -0.12 | -3.08 | 0.0020 | 18.25 | 8 | 0.0194 | 56.18 | 0.05 |  |  |  |  |
| **Lateral**  **occipital** | DSZ | 9 | -0.34 | 0.16 | 0.02 | -0.65 | -0.04 | -2.21 | 0.0270 | 21.20 | 8 | 0.0066 | 62.26 | 0.12 | Total between | 0.022 | 1 | 0.882 |
|  | NDSZ | 8 | -0.31 | 0.14 | 0.02 | -0.59 | -0.03 | -2.18 | 0.0289 | 31.72 | 7 | <0.0001 | 77.93 | 0.12 |  |  |  |  |
| **Lateral**  **orbitofrontal** | DSZ | 9 | -0.31 | 0.21 | 0.04 | -0.71 | 0.09 | -1.52 | 0.1295 | 37.80 | 8 | <0.0001 | 78.84 | 0.19 | Total between | 0.036 | 1 | 0.850 |
|  | NDSZ | 9 | -0.26 | 0.17 | 0.03 | -0.59 | 0.07 | -1.57 | 0.1173 | 47.60 | 8 | <0.0001 | 83.19 | 0.19 |  |  |  |  |
| **Lingual** | DSZ | 9 | -0.39 | 0.12 | 0.01 | -0.63 | -0.15 | -3.22 | 0.0013 | 13.31 | 8 | 0.1016 | 39.90 | 0.07 | Total between | 0.228 | 1 | 0.633 |
|  | NDSZ | 8 | -0.31 | 0.12 | 0.01 | -0.54 | -0.08 | -2.69 | 0.0072 | 20.89 | 7 | 0.0039 | 66.50 | 0.07 |  |  |  |  |
| **Medial orbito-frontal** | DSZ | 9 | -0.16 | 0.10 | 0.01 | -0.36 | 0.04 | -1.60 | 0.1091 | 9.81 | 8 | 0.2789 | 18.42 | 0.04 | Total between | 0.042 | 1 | 0.838 |
|  | NDSZ | 9 | -0.13 | 0.10 | 0.01 | -0.32 | 0.06 | -1.35 | 0.1765 | 16.37 | 8 | 0.0374 | 51.13 | 0.04 |  |  |  |  |
| **Middle**  **temporal** | DSZ | 9 | -0.49 | 0.13 | 0.02 | -0.75 | -0.23 | -3.66 | 0.0003 | 15.79 | 8 | 0.0454 | 49.35 | 0.07 | Total between | 0.971 | 1 | 0.324 |
|  | NDSZ | 9 | -0.32 | 0.12 | 0.01 | -0.54 | -0.09 | -2.73 | 0.0064 | 22.29 | 8 | 0.0044 | 64.11 | 0.07 |  |  |  |  |
| **Paracentral** | DSZ | 9 | -0.31 | 0.11 | 0.01 | -0.53 | -0.09 | -2.76 | 0.0057 | 11.70 | 8 | 0.1650 | 31.63 | 0.00 | Total between | 0.204 | 1 | 0.652 |
|  | NDSZ | 9 | -0.25 | 0.07 | 0.00 | -0.38 | -0.12 | -3.74 | 0.0002 | 8.66 | 8 | 0.3720 | 7.59 | 0.00 |  |  |  |  |
| **Para-hippocampal** | DSZ | 9 | -0.42 | 0.11 | 0.01 | -0.62 | -0.21 | -3.96 | <0.0001 | 10.50 | 8 | 0.2319 | 23.79 | 0.03 | Total between | 0.497 | 1 | 0.481 |
|  | NDSZ | 9 | -0.32 | 0.09 | 0.01 | -0.49 | -0.15 | -3.68 | 0.0002 | 13.19 | 8 | 0.1056 | 39.33 | 0.03 |  |  |  |  |
| **Pars**  **opercularis** | DSZ | 9 | -0.42 | 0.14 | 0.02 | -0.69 | -0.14 | -2.95 | 0.0032 | 17.60 | 8 | 0.0244 | 54.55 | 0.07 | Total between | 0.855 | 1 | 0.355 |
|  | NDSZ | 9 | -0.25 | 0.12 | 0.01 | -0.48 | -0.02 | -2.12 | 0.0340 | 23.02 | 8 | 0.0033 | 65.25 | 0.07 |  |  |  |  |
| **Pars**  **orbitalis** | DSZ | 9 | -0.42 | 0.10 | 0.01 | -0.62 | -0.22 | -4.14 | <0.0001 | 9.83 | 8 | 0.2773 | 18.60 | 0.04 | Total between | 1.443 | 1 | 0.230 |
|  | NDSZ | 9 | -0.25 | 0.10 | 0.01 | -0.44 | -0.06 | -2.61 | 0.0089 | 15.78 | 8 | 0.0457 | 49.29 | 0.04 |  |  |  |  |
| **Pars**  **triangularis** | DSZ | 9 | -0.39 | 0.10 | 0.01 | -0.59 | -0.19 | -3.89 | 0.0001 | 9.83 | 8 | 0.2772 | 18.61 | 0.02 | Total between | 0.920 | 1 | 0.337 |
|  | NDSZ | 9 | -0.27 | 0.08 | 0.01 | -0.43 | -0.11 | -3.26 | 0.0011 | 11.84 | 8 | 0.1585 | 32.43 | 0.02 |  |  |  |  |
|  | DSZ | 9 | -0.05 | 0.09 | 0.01 | -0.22 | 0.12 | -0.61 | 0.5403 | 3.01 | 8 | 0.9340 | 0.00 | 0.00 | Total between | 0.361 | 1 | 0.548 |
| **Pericalcarine** | NDSZ | 8 | 0.01 | 0.06 | 0.00 | -0.11 | 0.14 | 0.18 | 0.8549 | 6.69 | 7 | 0.4621 | 0.00 | 0.00 |  |  |  |  |
| **Postcentral** | DSZ | 9 | -0.46 | 0.15 | 0.02 | -0.75 | -0.17 | -3.11 | 0.0019 | 19.07 | 8 | 0.0145 | 58.05 | 0.07 | Total between | 0.825 | 1 | 0.364 |
|  | NDSZ | 9 | -0.29 | 0.12 | 0.01 | -0.51 | -0.06 | -2.50 | 0.0126 | 22.37 | 8 | 0.0043 | 64.24 | 0.07 |  |  |  |  |
| **Posterior**  **cingulate** | DSZ | 9 | -0.36 | 0.11 | 0.01 | -0.56 | -0.15 | -3.33 | 0.0009 | 10.76 | 8 | 0.2157 | 25.65 | 0.06 | Total between | 0.434 | 1 | 0.510 |
|  | NDSZ | 9 | -0.25 | 0.11 | 0.01 | -0.47 | -0.04 | -2.31 | 0.0206 | 20.56 | 8 | 0.0084 | 61.10 | 0.06 |  |  |  |  |
| **Precentral** | DSZ | 9 | -0.46 | 0.12 | 0.01 | -0.69 | -0.23 | -3.95 | <0.0001 | 12.34 | 8 | 0.1366 | 35.17 | 0.05 | Total between | 1.767 | 1 | 0.184 |
|  | NDSZ | 9 | -0.25 | 0.11 | 0.01 | -0.46 | -0.04 | -2.38 | 0.0172 | 18.84 | 8 | 0.0157 | 57.54 | 0.05 |  |  |  |  |
| **Precuneus** | DSZ | 9 | -0.40 | 0.10 | 0.01 | -0.59 | -0.21 | -4.07 | <0.0001 | 9.41 | 8 | 0.3087 | 15.01 | 0.05 | Total between | 1.222 | 1 | 0.269 |
|  | NDSZ | 9 | -0.24 | 0.11 | 0.01 | -0.45 | -0.03 | -2.23 | 0.0259 | 19.37 | 8 | 0.0130 | 58.70 | 0.05 |  |  |  |  |
| **Rostral**  **anterior**  **cingulate** | DSZ | 9 | -0.16 | 0.13 | 0.02 | -0.41 | 0.09 | -1.27 | 0.2034 | 14.44 | 8 | 0.0710 | 44.60 | 0.09 | Total between | 0.000 | 1 | 0.992 |
|  | NDSZ | 9 | -0.16 | 0.12 | 0.02 | -0.40 | 0.08 | -1.32 | 0.1865 | 25.74 | 8 | 0.0012 | 68.92 | 0.09 |  |  |  |  |
| **Rostral**  **middle**  **frontal** | DSZ | 9 | -0.34 | 0.10 | 0.01 | -0.54 | -0.14 | -3.38 | 0.0007 | 9.84 | 8 | 0.2764 | 18.71 | 0.04 | Total between | 0.971 | 1 | 0.325 |
|  | NDSZ | 9 | -0.20 | 0.10 | 0.01 | -0.39 | -0.01 | -2.10 | 0.0361 | 16.11 | 8 | 0.0408 | 50.35 | 0.04 |  |  |  |  |
| **Superior**  **frontal** | DSZ | 9 | -0.58 | 0.13 | 0.02 | -0.84 | -0.32 | -4.41 | <0.0001 | 15.48 | 8 | 0.0505 | 48.32 | 0.05 | Total between | 0.954 | 1 | 0.329 |
|  | NDSZ | 9 | -0.42 | 0.10 | 0.01 | -0.62 | -0.22 | -4.08 | <0.0001 | 17.93 | 8 | 0.0218 | 55.37 | 0.05 |  |  |  |  |
| **Superior**  **parietal** | DSZ | 9 | -0.33 | 0.09 | 0.01 | -0.50 | -0.16 | -3.86 | 0.0001 | 6.09 | 8 | 0.6366 | 0.00 | 0.01 | Total between | 0.695 | 1 | 0.404 |
|  | NDSZ | 9 | -0.24 | 0.08 | 0.01 | -0.39 | -0.08 | -3.06 | 0.0022 | 10.62 | 8 | 0.2242 | 24.67 | 0.01 |  |  |  |  |
| **Superior**  **temporal** | DSZ | 9 | -0.52 | 0.15 | 0.02 | -0.80 | -0.23 | -3.55 | 0.0004 | 18.03 | 8 | 0.0210 | 55.64 | 0.10 | Total between | 0.811 | 1 | 0.368 |
|  | NDSZ | 9 | -0.34 | 0.13 | 0.02 | -0.60 | -0.09 | -2.63 | 0.0086 | 27.75 | 8 | 0.0005 | 71.18 | 0.10 |  |  |  |  |
| **Supramarginal** | DSZ | 9 | -0.60 | 0.13 | 0.02 | -0.85 | -0.34 | -4.58 | <0.0001 | 14.98 | 8 | 0.0596 | 46.58 | 0.04 | Total between | 0.503 | 1 | 0.478 |
|  | NDSZ | 9 | -0.48 | 0.10 | 0.01 | -0.67 | -0.29 | -4.97 | <0.0001 | 15.80 | 8 | 0.0453 | 49.37 | 0.04 |  |  |  |  |
| **Temporal pole** | DSZ | 9 | -0.32 | 0.13 | 0.02 | -0.58 | -0.06 | -2.45 | 0.0141 | 15.24 | 8 | 0.0546 | 47.51 | 0.06 | Total between | 0.232 | 1 | 0.630 |
|  | NDSZ | 9 | -0.24 | 0.11 | 0.01 | -0.45 | -0.02 | -2.16 | 0.0309 | 20.69 | 8 | 0.0080 | 61.34 | 0.06 |  |  |  |  |
| **Transverse**  **temporal** | DSZ | 9 | -0.30 | 0.13 | 0.02 | -0.55 | -0.04 | -2.30 | 0.0213 | 14.71 | 8 | 0.0651 | 45.60 | 0.03 | Total between | 0.685 | 1 | 0.408 |
|  | NDSZ | 9 | -0.17 | 0.09 | 0.01 | -0.34 | 0.00 | -1.90 | 0.0570 | 13.39 | 8 | 0.0990 | 40.26 | 0.03 |  |  |  |  |
| **Mean thickness** | DSZ | 9 | -0.62 | 0.15 | 0.02 | -0.90 | -0.33 | -4.26 | <0.0001 | 18.40 | 8 | 0.0184 | 56.52 | 0.12 | Total between | 0.667 | 1 | 0.414 |
|  | NDSZ | 9 | -0.44 | 0.14 | 0.02 | -0.71 | -0.16 | -3.15 | 0.0016 | 32.49 | 8 | <0.0001 | 75.38 | 0.12 |  |  |  |  |

**Legend:** *DSZ, Deficit Schizophrenia; NDSZ, Non Deficit Schizophrenia; Point estim, estimated standard difference; Std err, Standard Error; Var, Variance; df, degrees of freedom; Sqrd, Squared.*

**Supplementary Table S9**. Results from random effect Meta-Analysis of left cortical surface area

| **Cortical**  **Regions** | **Groups** | **Effect size and 95% confidence interval** | | | | | | **Test of null**  **(2-Tail)** | | **Heterogeneity** | | | | **Sub-groups Analysis** | | | | |
| --- | --- | --- | --- | --- | --- | --- | --- | --- | --- | --- | --- | --- | --- | --- | --- | --- | --- | --- |
|  |  | **N** | **Point estim** | **Std err** | **Var** | **Low limit** | **Up limit** | **Z-value** | ***P-***  ***value*** | **Q-value** | **df** | ***P-value*** | **I-**  **sqrd** | **Tau- Sqrd** |  | **Q-value** | **df** | ***P-value*** |
| **Banks sts** | DSZ | 9 | -0.07 | 0.09 | 0.01 | -0.24 | 0.10 | -0.79 | 0.431 | 5.57 | 8 | 0.696 | 0.00 | 0.00 | Total between | 0.347 | 1 | 0.556 |
|  | NDSZ | 9 | 0.00 | 0.07 | 0.01 | -0.14 | 0.14 | -0.02 | 0.983 | 9.65 | 8 | 0.291 | 17.08 | 0.01 |  |  |  |  |
| **Caudal**  **anterior**  **cingulate** | DSZ | 9 | -0.11 | 0.09 | 0.01 | -0.28 | 0.06 | -1.30 | 0.194 | 7.47 | 8 | 0.487 | 0.00 | 0.00 | Total between | 0.239 | 1 | 0.625 |
|  | NDSZ | 9 | -0.17 | 0.07 | 0.01 | -0.31 | -0.03 | -2.33 | 0.020 | 9.44 | 8 | 0.307 | 15.26 | 0.01 |  |  |  |  |
| **Caudal**  **middle**  **frontal** | DSZ | 9 | -0.08 | 0.09 | 0.01 | -0.25 | 0.09 | -0.93 | 0.354 | 5.24 | 8 | 0.732 | 0.00 | 0.00 | Total between | 0.162 | 1 | 0.688 |
|  | NDSZ | 9 | -0.03 | 0.08 | 0.01 | -0.19 | 0.12 | -0.42 | 0.674 | 10.98 | 8 | 0.203 | 27.12 | 0.01 |  |  |  |  |
| **Cuneus** | DSZ | 9 | -0.09 | 0.09 | 0.01 | -0.26 | 0.07 | -1.10 | 0.273 | 6.24 | 8 | 0.621 | 0.00 | 0.00 | Total between | 0.019 | 1 | 0.892 |
|  | NDSZ | 9 | -0.11 | 0.07 | 0.01 | -0.25 | 0.03 | -1.51 | 0.130 | 9.58 | 8 | 0.295 | 16.53 | 0.01 |  |  |  |  |
| **Entorhinal** | DSZ | 9 | -0.17 | 0.09 | 0.01 | -0.34 | 0.00 | -1.96 | 0.050 | 2.90 | 8 | 0.941 | 0.00 | 0.00 | Total between | 0.106 | 1 | 0.745 |
|  | NDSZ | 9 | -0.13 | 0.08 | 0.01 | -0.28 | 0.02 | -1.73 | 0.083 | 10.33 | 8 | 0.243 | 22.57 | 0.01 |  |  |  |  |
| **Frontal pole** | DSZ | 9 | -0.02 | 0.13 | 0.02 | -0.28 | 0.25 | -0.11 | 0.909 | 15.94 | 8 | 0.043 | 49.82 | 0.07 | Total between | 0.062 | 1 | 0.804 |
|  | NDSZ | 9 | 0.03 | 0.10 | 0.01 | -0.17 | 0.23 | 0.26 | 0.795 | 17.64 | 8 | 0.024 | 54.66 | 0.05 |  |  |  |  |
| **Fusiform** | DSZ | 9 | -0.27 | 0.09 | 0.01 | -0.45 | -0.10 | -3.00 | 0.003 | 8.65 | 8 | 0.373 | 7.50 | 0.01 | Total between | 1.189 | 1 | 0.276 |
|  | NDSZ | 9 | -0.13 | 0.09 | 0.01 | -0.31 | 0.05 | -1.45 | 0.147 | 14.53 | 8 | 0.069 | 44.95 | 0.03 |  |  |  |  |
| **Inferior**  **parietal** | DSZ | 9 | -0.20 | 0.15 | 0.02 | -0.50 | 0.09 | -1.36 | 0.175 | 20.19 | 8 | 0.010 | 60.38 | 0.11 | Total between | 0.938 | 1 | 0.333 |
|  | NDSZ | 9 | -0.02 | 0.12 | 0.01 | -0.25 | 0.21 | -0.17 | 0.866 | 23.16 | 8 | 0.003 | 65.46 | 0.07 |  |  |  |  |
| **Inferior**  **temporal** | DSZ | 9 | -0.14 | 0.14 | 0.02 | -0.42 | 0.14 | -0.98 | 0.328 | 17.78 | 8 | 0.023 | 55.00 | 0.09 | Total between | 0.039 | 1 | 0.844 |
|  | NDSZ | 9 | -0.10 | 0.10 | 0.01 | -0.30 | 0.09 | -1.06 | 0.289 | 16.62 | 8 | 0.034 | 51.86 | 0.04 |  |  |  |  |
| **Insula** | DSZ | 9 | -0.09 | 0.10 | 0.01 | -0.29 | 0.11 | -0.89 | 0.372 | 10.26 | 8 | 0.247 | 22.01 | 0.02 | Total between | 0.498 | 1 | 0.480 |
|  | NDSZ | 9 | 0.00 | 0.07 | 0.01 | -0.15 | 0.14 | -0.04 | 0.972 | 9.96 | 8 | 0.268 | 19.71 | 0.01 |  |  |  |  |
| **Isthmus**  **cingulate** | DSZ | 9 | -0.03 | 0.11 | 0.01 | -0.25 | 0.18 | -0.30 | 0.766 | 11.29 | 8 | 0.186 | 29.16 | 0.03 | Total between | 0.413 | 1 | 0.521 |
|  | NDSZ | 9 | 0.06 | 0.09 | 0.01 | -0.12 | 0.24 | 0.64 | 0.520 | 14.93 | 8 | 0.060 | 46.43 | 0.03 |  |  |  |  |
| **Lateral**  **occipital** | DSZ | 9 | -0.15 | 0.10 | 0.01 | -0.34 | 0.04 | -1.58 | 0.113 | 9.23 | 8 | 0.324 | 13.29 | 0.01 | Total between | 0.197 | 1 | 0.657 |
|  | NDSZ | 9 | -0.09 | 0.10 | 0.01 | -0.29 | 0.11 | -0.89 | 0.372 | 17.15 | 8 | 0.029 | 53.36 | 0.04 |  |  |  |  |
| **Lateral**  **orbitofrontal** | DSZ | 9 | -0.19 | 0.17 | 0.03 | -0.53 | 0.15 | -1.08 | 0.281 | 27.09 | 8 | 0.001 | 70.47 | 0.17 | Total between | 0.189 | 1 | 0.664 |
|  | NDSZ | 9 | -0.09 | 0.14 | 0.02 | -0.36 | 0.17 | -0.68 | 0.497 | 31.04 | 8 | 0.000 | 74.23 | 0.11 |  |  |  |  |
| **Lingual** | DSZ | 9 | -0.12 | 0.09 | 0.01 | -0.29 | 0.04 | -1.46 | 0.145 | 5.36 | 8 | 0.719 | 0.00 | 0.00 | Total between | 0.612 | 1 | 0.434 |
|  | NDSZ | 9 | -0.04 | 0.08 | 0.01 | -0.18 | 0.11 | -0.46 | 0.646 | 10.52 | 8 | 0.230 | 23.95 | 0.01 |  |  |  |  |
| **Medial orbito-frontal** | DSZ | 9 | -0.07 | 0.15 | 0.02 | -0.36 | 0.21 | -0.51 | 0.610 | 18.63 | 8 | 0.017 | 57.06 | 0.10 | Total between | 0.242 | 1 | 0.623 |
|  | NDSZ | 9 | 0.02 | 0.12 | 0.02 | -0.22 | 0.26 | 0.16 | 0.874 | 25.85 | 8 | 0.001 | 69.06 | 0.09 |  |  |  |  |
| **Middle**  **temporal** | DSZ | 9 | -0.14 | 0.14 | 0.02 | -0.41 | 0.12 | -1.05 | 0.293 | 15.90 | 8 | 0.044 | 49.69 | 0.07 | Total between | 0.155 | 1 | 0.694 |
|  | NDSZ | 9 | -0.07 | 0.11 | 0.01 | -0.29 | 0.14 | -0.67 | 0.503 | 20.24 | 8 | 0.009 | 60.47 | 0.06 |  |  |  |  |
| **Paracentral** | DSZ | 9 | -0.03 | 0.09 | 0.01 | -0.20 | 0.14 | -0.38 | 0.707 | 5.35 | 8 | 0.720 | 0.00 | 0.00 | Total between | 0.129 | 1 | 0.719 |
|  | NDSZ | 9 | 0.01 | 0.06 | 0.00 | -0.12 | 0.13 | 0.10 | 0.922 | 4.91 | 8 | 0.767 | 0.00 | 0.00 |  |  |  |  |
| **Para-hippocampal** | DSZ | 9 | 0.00 | 0.10 | 0.01 | -0.19 | 0.19 | 0.01 | 0.988 | 9.49 | 8 | 0.303 | 15.67 | 0.01 | Total between | 0.082 | 1 | 0.775 |
|  | NDSZ | 9 | 0.04 | 0.08 | 0.01 | -0.12 | 0.19 | 0.48 | 0.634 | 11.00 | 8 | 0.201 | 27.31 | 0.01 |  |  |  |  |
| **Pars**  **opercularis** | DSZ | 9 | -0.08 | 0.09 | 0.01 | -0.24 | 0.09 | -0.88 | 0.380 | 7.45 | 8 | 0.489 | 0.00 | 0.00 | Total between | 0.540 | 1 | 0.462 |
|  | NDSZ | 9 | 0.02 | 0.10 | 0.01 | -0.17 | 0.21 | 0.20 | 0.842 | 15.62 | 8 | 0.048 | 48.78 | 0.04 |  |  |  |  |
| **Pars**  **orbitalis** | DSZ | 9 | -0.20 | 0.13 | 0.02 | -0.45 | 0.05 | -1.59 | 0.111 | 14.18 | 8 | 0.077 | 43.57 | 0.06 | Total between | 0.847 | 1 | 0.357 |
|  | NDSZ | 9 | -0.04 | 0.13 | 0.02 | -0.28 | 0.21 | -0.28 | 0.777 | 27.21 | 8 | 0.001 | 70.60 | 0.09 |  |  |  |  |
| **Pars**  **triangularis** | DSZ | 9 | -0.19 | 0.09 | 0.01 | -0.36 | -0.03 | -2.26 | 0.024 | 7.55 | 8 | 0.478 | 0.00 | 0.00 | Total between | 1.309 | 1 | 0.253 |
|  | NDSZ | 9 | -0.06 | 0.08 | 0.01 | -0.21 | 0.08 | -0.85 | 0.395 | 10.25 | 8 | 0.248 | 21.96 | 0.01 |  |  |  |  |
|  | DSZ | 9 | -0.11 | 0.09 | 0.01 | -0.27 | 0.06 | -1.25 | 0.212 | 4.06 | 8 | 0.852 | 0.00 | 0.00 | Total between | 0.085 | 1 | 0.771 |
| **Pericalcarine** | NDSZ | 9 | -0.07 | 0.11 | 0.01 | -0.27 | 0.14 | -0.63 | 0.525 | 19.09 | 8 | 0.014 | 58.09 | 0.05 |  |  |  |  |
| **Postcentral** | DSZ | 9 | -0.15 | 0.09 | 0.01 | -0.32 | 0.02 | -1.72 | 0.085 | 6.23 | 8 | 0.621 | 0.00 | 0.00 | Total between | 1.350 | 1 | 0.245 |
|  | NDSZ | 9 | -0.02 | 0.06 | 0.00 | -0.15 | 0.10 | -0.38 | 0.707 | 7.57 | 8 | 0.476 | 0.00 | 0.00 |  |  |  |  |
| **Posterior**  **cingulate** | DSZ | 9 | -0.04 | 0.09 | 0.01 | -0.20 | 0.13 | -0.42 | 0.674 | 7.80 | 8 | 0.454 | 0.00 | 0.00 | Total between | 0.008 | 1 | 0.930 |
|  | NDSZ | 9 | -0.05 | 0.07 | 0.01 | -0.19 | 0.09 | -0.64 | 0.520 | 9.45 | 8 | 0.306 | 15.37 | 0.01 |  |  |  |  |
| **Precentral** | DSZ | 9 | -0.11 | 0.10 | 0.01 | -0.31 | 0.09 | -1.08 | 0.280 | 10.00 | 8 | 0.265 | 20.02 | 0.02 | Total between | 0.043 | 1 | 0.835 |
|  | NDSZ | 9 | -0.08 | 0.10 | 0.01 | -0.28 | 0.12 | -0.79 | 0.430 | 17.44 | 8 | 0.026 | 54.13 | 0.05 |  |  |  |  |
| **Precuneus** | DSZ | 9 | -0.03 | 0.09 | 0.01 | -0.20 | 0.14 | -0.36 | 0.716 | 5.15 | 8 | 0.742 | 0.00 | 0.00 | Total between | 0.074 | 1 | 0.785 |
|  | NDSZ | 9 | 0.00 | 0.06 | 0.00 | -0.13 | 0.12 | -0.03 | 0.973 | 6.67 | 8 | 0.573 | 0.00 | 0.00 |  |  |  |  |
| **Rostral**  **anterior**  **cingulate** | DSZ | 9 | -0.06 | 0.12 | 0.01 | -0.29 | 0.17 | -0.48 | 0.630 | 12.55 | 8 | 0.128 | 36.27 | 0.04 | Total between | 0.000 | 1 | 1.000 |
|  | NDSZ | 9 | -0.06 | 0.09 | 0.01 | -0.24 | 0.12 | -0.61 | 0.539 | 14.68 | 8 | 0.066 | 45.50 | 0.03 |  |  |  |  |
| **Rostral**  **middle**  **frontal** | DSZ | 9 | -0.16 | 0.11 | 0.01 | -0.37 | 0.05 | -1.49 | 0.136 | 10.99 | 8 | 0.203 | 27.18 | 0.03 | Total between | 0.570 | 1 | 0.450 |
|  | NDSZ | 9 | -0.05 | 0.10 | 0.01 | -0.24 | 0.14 | -0.53 | 0.599 | 16.20 | 8 | 0.040 | 50.63 | 0.04 |  |  |  |  |
| **Superior**  **frontal** | DSZ | 9 | -0.20 | 0.09 | 0.01 | -0.36 | -0.03 | -2.28 | 0.022 | 7.75 | 8 | 0.459 | 0.00 | 0.00 | Total between | 1.087 | 1 | 0.297 |
|  | NDSZ | 9 | -0.06 | 0.10 | 0.01 | -0.25 | 0.13 | -0.62 | 0.535 | 16.31 | 8 | 0.038 | 50.95 | 0.04 |  |  |  |  |
| **Superior**  **parietal** | DSZ | 9 | -0.11 | 0.09 | 0.01 | -0.28 | 0.06 | -1.31 | 0.190 | 2.06 | 8 | 0.979 | 0.00 | 0.00 | Total between | 1.115 | 1 | 0.291 |
|  | NDSZ | 9 | 0.00 | 0.06 | 0.00 | -0.12 | 0.12 | 0.00 | 0.998 | 4.75 | 8 | 0.784 | 0.00 | 0.00 |  |  |  |  |
| **Superior**  **temporal** | DSZ | 9 | -0.17 | 0.09 | 0.01 | -0.34 | 0.00 | -1.94 | 0.053 | 7.19 | 8 | 0.516 | 0.00 | 0.00 | Total between | 0.598 | 1 | 0.439 |
|  | NDSZ | 9 | -0.07 | 0.09 | 0.01 | -0.24 | 0.09 | -0.87 | 0.387 | 12.29 | 8 | 0.139 | 34.90 | 0.02 |  |  |  |  |
| **Supramarginal** | DSZ | 9 | -0.03 | 0.11 | 0.01 | -0.25 | 0.19 | -0.29 | 0.770 | 11.58 | 8 | 0.171 | 30.94 | 0.03 | Total between | 0.083 | 1 | 0.774 |
|  | NDSZ | 9 | 0.01 | 0.12 | 0.01 | -0.22 | 0.25 | 0.12 | 0.906 | 23.73 | 8 | 0.003 | 66.29 | 0.08 |  |  |  |  |
| **Temporal pole** | DSZ | 9 | 0.07 | 0.09 | 0.01 | -0.10 | 0.24 | 0.79 | 0.430 | 4.37 | 8 | 0.822 | 0.00 | 0.00 | Total between | 0.005 | 1 | 0.943 |
|  | NDSZ | 9 | 0.06 | 0.07 | 0.01 | -0.08 | 0.20 | 0.81 | 0.420 | 9.97 | 8 | 0.267 | 19.76 | 0.01 |  |  |  |  |
| **Transverse**  **temporal** | DSZ | 9 | -0.10 | 0.10 | 0.01 | -0.30 | 0.10 | -0.98 | 0.325 | 10.10 | 8 | 0.258 | 20.79 | 0.02 | Total between | 0.195 | 1 | 0.659 |
|  | NDSZ | 9 | -0.04 | 0.08 | 0.01 | -0.21 | 0.12 | -0.50 | 0.616 | 12.46 | 8 | 0.132 | 35.78 | 0.02 |  |  |  |  |
| **Total Surface** | DSZ | 9 | -0.26 | 0.14 | 0.02 | -0.53 | 0.01 | -1.87 | 0.062 | 16.93 | 8 | 0.031 | 52.74 | 0.08 | Total between | 0.561 | 1 | 0.454 |
|  | NDSZ | 9 | -0.12 | 0.12 | 0.01 | -0.35 | 0.11 | -1.05 | 0.295 | 23.09 | 8 | 0.003 | 65.36 | 0.07 |  |  |  |  |

**Legend:** *DSZ, Deficit Schizophrenia; NDSZ, Non Deficit Schizophrenia; Point estim, estimated standard difference; Std err, Standard Error; Var, Variance; df, degrees of freedom; Sqrd, Squared*

**Supplementary Table S10**. Results from random effect Meta-Analysis of right cortical surface area

| **Cortical**  **Regions** | **Groups** | **Effect size and 95% confidence interval** | | | | | | **Test of null**  **(2-Tail)** | | **Heterogeneity** | | | | **Sub-groups Analysis** | | | | |
| --- | --- | --- | --- | --- | --- | --- | --- | --- | --- | --- | --- | --- | --- | --- | --- | --- | --- | --- |
|  |  | **N** | **Point estim** | **Std err** | **Var** | **Low limit** | **Up limit** | **Z-value** | ***P-***  ***value*** | **Q-value** | **df** | ***P-value*** | **I-**  **sqrd** | **Tau- Sqrd** |  | **Q-value** | **df** | ***P-value*** |
| **Bankssts** | DSZ | 9 | -0.10 | 0.12 | 0.02 | -0.35 | 0.14 | -0.84 | 0.4005 | 13.72 | 8 | 0.089 | 41.70 | 0.05 | Total between | 0.085 | 1 | 0.770 |
|  | NDSZ | 9 | -0.06 | 0.11 | 0.01 | -0.27 | 0.16 | -0.51 | 0.6076 | 20.07 | 8 | 0.010 | 60.15 | 0.06 |  |  |  |  |
| **Caudal**  **anterior**  **cingulate** | DSZ | 9 | -0.23 | 0.12 | 0.01 | -0.45 | 0.00 | -1.94 | 0.0527 | 12.39 | 8 | 0.134 | 35.46 | 0.04 | Total between | 0.029 | 1 | 0.864 |
|  | NDSZ | 9 | -0.20 | 0.06 | 0.00 | -0.33 | -0.08 | -3.19 | 0.0014 | 6.94 | 8 | 0.543 | 0.00 | 0.00 |  |  |  |  |
| **Caudal**  **middle**  **frontal** | DSZ | 9 | -0.14 | 0.13 | 0.02 | -0.39 | 0.11 | -1.10 | 0.2732 | 14.60 | 8 | 0.067 | 45.20 | 0.06 | Total between | 0.255 | 1 | 0.614 |
|  | NDSZ | 9 | -0.05 | 0.11 | 0.01 | -0.27 | 0.16 | -0.50 | 0.6175 | 20.34 | 8 | 0.009 | 60.68 | 0.06 |  |  |  |  |
| **Cuneus** | DSZ | 9 | -0.14 | 0.13 | 0.02 | -0.39 | 0.11 | -1.08 | 0.2792 | 14.48 | 8 | 0.070 | 44.76 | 0.06 | Total between | 0.531 | 1 | 0.466 |
|  | NDSZ | 9 | -0.02 | 0.10 | 0.01 | -0.22 | 0.18 | -0.20 | 0.8450 | 17.37 | 8 | 0.027 | 53.94 | 0.04 |  |  |  |  |
| **Entorhinal** | DSZ | 9 | -0.09 | 0.09 | 0.01 | -0.26 | 0.08 | -1.08 | 0.2803 | 8.13 | 8 | 0.421 | 1.56 | 0.00 | Total between | 0.020 | 1 | 0.888 |
|  | NDSZ | 9 | -0.08 | 0.08 | 0.01 | -0.24 | 0.09 | -0.91 | 0.3651 | 12.58 | 8 | 0.127 | 36.38 | 0.02 |  |  |  |  |
| **Frontal pole** | DSZ | 9 | 0.02 | 0.12 | 0.01 | -0.22 | 0.26 | 0.16 | 0.8759 | 13.63 | 8 | 0.092 | 41.32 | 0.05 | Total between | 0.030 | 1 | 0.861 |
|  | NDSZ | 9 | -0.01 | 0.08 | 0.01 | -0.17 | 0.16 | -0.08 | 0.9355 | 12.56 | 8 | 0.128 | 36.28 | 0.02 |  |  |  |  |
| **Fusiform** | DSZ | 9 | -0.15 | 0.12 | 0.02 | -0.39 | 0.09 | -1.19 | 0.2332 | 13.82 | 8 | 0.087 | 42.11 | 0.05 | Total between | 0.138 | 1 | 0.710 |
|  | NDSZ | 9 | -0.09 | 0.11 | 0.01 | -0.30 | 0.13 | -0.79 | 0.4284 | 20.04 | 8 | 0.010 | 60.09 | 0.06 |  |  |  |  |
| **Inferior**  **parietal** | DSZ | 9 | -0.12 | 0.15 | 0.02 | -0.42 | 0.18 | -0.78 | 0.4351 | 20.76 | 8 | 0.008 | 61.47 | 0.11 | Total between | 0.156 | 1 | 0.693 |
|  | NDSZ | 9 | -0.04 | 0.11 | 0.01 | -0.26 | 0.17 | -0.40 | 0.6855 | 20.74 | 8 | 0.008 | 61.44 | 0.06 |  |  |  |  |
| **Inferior**  **temporal** | DSZ | 9 | -0.18 | 0.11 | 0.01 | -0.41 | 0.04 | -1.60 | 0.1093 | 11.94 | 8 | 0.154 | 33.02 | 0.04 | Total between | 0.927 | 1 | 0.336 |
|  | NDSZ | 9 | -0.03 | 0.10 | 0.01 | -0.24 | 0.17 | -0.34 | 0.7362 | 17.94 | 8 | 0.022 | 55.40 | 0.05 |  |  |  |  |
| **Insula** | DSZ | 9 | -0.12 | 0.11 | 0.01 | -0.34 | 0.10 | -1.07 | 0.2853 | 11.45 | 8 | 0.178 | 30.10 | 0.03 | Total between | 0.319 | 1 | 0.572 |
|  | NDSZ | 9 | -0.04 | 0.08 | 0.01 | -0.19 | 0.11 | -0.54 | 0.5883 | 10.82 | 8 | 0.212 | 26.05 | 0.01 |  |  |  |  |
| **Isthmus**  **cingulate** | DSZ | 9 | 0.02 | 0.10 | 0.01 | -0.17 | 0.21 | 0.21 | 0.8356 | 9.17 | 8 | 0.328 | 12.76 | 0.01 | Total between | 1.072 | 1 | 0.300 |
|  | NDSZ | 9 | 0.14 | 0.07 | 0.01 | 0.00 | 0.28 | 2.01 | 0.0440 | 9.40 | 8 | 0.309 | 14.92 | 0.01 |  |  |  |  |
| **Lateral**  **occipital** | DSZ | 9 | -0.08 | 0.09 | 0.01 | -0.26 | 0.10 | -0.89 | 0.3714 | 8.79 | 8 | 0.360 | 8.98 | 0.01 | Total between | 0.211 | 1 | 0.646 |
|  | NDSZ | 9 | -0.03 | 0.08 | 0.01 | -0.19 | 0.14 | -0.30 | 0.7653 | 11.29 | 7 | 0.126 | 38.01 | 0.02 |  |  |  |  |
| **Lateral**  **orbitofrontal** | DSZ | 9 | -0.07 | 0.15 | 0.02 | -0.36 | 0.22 | -0.45 | 0.6544 | 19.45 | 8 | 0.013 | 58.87 | 0.10 | Total between | 0.139 | 1 | 0.709 |
|  | NDSZ | 9 | 0.00 | 0.12 | 0.01 | -0.23 | 0.23 | 0.04 | 0.9710 | 23.51 | 8 | 0.003 | 65.98 | 0.07 |  |  |  |  |
| **Lingual** | DSZ | 9 | -0.09 | 0.09 | 0.01 | -0.26 | 0.08 | -1.07 | 0.2865 | 4.02 | 8 | 0.856 | 0.00 | 0.00 | Total between | 0.329 | 1 | 0.566 |
|  | NDSZ | 9 | -0.03 | 0.08 | 0.01 | -0.18 | 0.13 | -0.33 | 0.7418 | 9.52 | 7 | 0.217 | 26.48 | 0.01 |  |  |  |  |
| **Medial orbito-frontal** | DSZ | 9 | -0.09 | 0.13 | 0.02 | -0.34 | 0.16 | -0.72 | 0.4710 | 14.49 | 8 | 0.070 | 44.80 | 0.06 | Total between | 0.011 | 1 | 0.916 |
|  | NDSZ | 9 | -0.07 | 0.09 | 0.01 | -0.25 | 0.10 | -0.85 | 0.3953 | 13.54 | 8 | 0.094 | 40.93 | 0.03 |  |  |  |  |
| **Middle**  **temporal** | DSZ | 9 | -0.06 | 0.11 | 0.01 | -0.28 | 0.17 | -0.52 | 0.6062 | 12.17 | 8 | 0.144 | 34.28 | 0.04 | Total between | 0.010 | 1 | 0.922 |
|  | NDSZ | 9 | -0.04 | 0.10 | 0.01 | -0.23 | 0.14 | -0.47 | 0.6416 | 15.60 | 8 | 0.048 | 48.72 | 0.04 |  |  |  |  |
| **Paracentral** | DSZ | 9 | -0.12 | 0.09 | 0.01 | -0.29 | 0.04 | -1.46 | 0.1446 | 3.42 | 8 | 0.905 | 0.00 | 0.00 | Total between | 0.244 | 1 | 0.621 |
|  | NDSZ | 9 | -0.07 | 0.06 | 0.00 | -0.20 | 0.05 | -1.14 | 0.2526 | 5.06 | 8 | 0.751 | 0.00 | 0.00 |  |  |  |  |
| **Para-hippocampal** | DSZ | 9 | 0.04 | 0.09 | 0.01 | -0.13 | 0.22 | 0.51 | 0.6083 | 8.18 | 8 | 0.416 | 2.15 | 0.00 | Total between | 0.103 | 1 | 0.749 |
|  | NDSZ | 9 | 0.01 | 0.09 | 0.01 | -0.16 | 0.17 | 0.07 | 0.9465 | 12.72 | 8 | 0.122 | 37.11 | 0.02 |  |  |  |  |
| **Pars**  **opercularis** | DSZ | 9 | -0.13 | 0.12 | 0.01 | -0.37 | 0.10 | -1.13 | 0.2595 | 12.81 | 8 | 0.119 | 37.54 | 0.04 | Total between | 0.310 | 1 | 0.578 |
|  | NDSZ | 9 | -0.05 | 0.09 | 0.01 | -0.22 | 0.12 | -0.58 | 0.5610 | 13.58 | 8 | 0.093 | 41.09 | 0.03 |  |  |  |  |
| **Pars**  **orbitalis** | DSZ | 9 | -0.17 | 0.09 | 0.01 | -0.34 | 0.00 | -1.96 | 0.0500 | 5.74 | 8 | 0.676 | 0.00 | 0.00 | Total between | 0.136 | 1 | 0.713 |
|  | NDSZ | 9 | -0.13 | 0.08 | 0.01 | -0.28 | 0.02 | -1.64 | 0.1003 | 10.59 | 8 | 0.226 | 24.43 | 0.01 |  |  |  |  |
| **Pars**  **triangularis** | DSZ | 9 | -0.13 | 0.09 | 0.01 | -0.30 | 0.04 | -1.54 | 0.1242 | 4.27 | 8 | 0.832 | 0.00 | 0.00 | Total between | 0.215 | 1 | 0.643 |
|  | NDSZ | 9 | -0.08 | 0.08 | 0.01 | -0.23 | 0.08 | -1.00 | 0.3182 | 10.96 | 8 | 0.204 | 26.98 | 0.01 |  |  |  |  |
|  | DSZ | 9 | -0.12 | 0.12 | 0.01 | -0.35 | 0.12 | -0.95 | 0.3429 | 13.31 | 8 | 0.102 | 39.90 | 0.05 | Total between | 0.264 | 1 | 0.607 |
| **Pericalcarine** | NDSZ | 9 | -0.04 | 0.09 | 0.01 | -0.22 | 0.15 | -0.38 | 0.7026 | 13.87 | 7 | 0.054 | 49.52 | 0.03 |  |  |  |  |
| **Postcentral** | DSZ | 9 | -0.11 | 0.10 | 0.01 | -0.30 | 0.07 | -1.18 | 0.2385 | 9.19 | 8 | 0.327 | 12.94 | 0.01 | Total between | 0.200 | 1 | 0.655 |
|  | NDSZ | 9 | -0.06 | 0.08 | 0.01 | -0.22 | 0.10 | -0.69 | 0.4914 | 11.90 | 8 | 0.156 | 32.76 | 0.02 |  |  |  |  |
| **Posterior**  **cingulate** | DSZ | 9 | -0.14 | 0.12 | 0.01 | -0.37 | 0.09 | -1.19 | 0.2338 | 12.47 | 8 | 0.131 | 35.84 | 0.04 | Total between | 0.316 | 1 | 0.574 |
|  | NDSZ | 9 | -0.06 | 0.09 | 0.01 | -0.23 | 0.12 | -0.61 | 0.5431 | 14.45 | 8 | 0.071 | 44.63 | 0.03 |  |  |  |  |
| **Precentral** | DSZ | 9 | -0.18 | 0.10 | 0.01 | -0.38 | 0.01 | -1.87 | 0.0615 | 9.51 | 8 | 0.301 | 15.91 | 0.01 | Total between | 0.008 | 1 | 0.931 |
|  | NDSZ | 9 | -0.17 | 0.09 | 0.01 | -0.34 | 0.00 | -1.98 | 0.0482 | 13.20 | 8 | 0.105 | 39.40 | 0.03 |  |  |  |  |
| **Precuneus** | DSZ | 9 | -0.11 | 0.09 | 0.01 | -0.28 | 0.06 | -1.27 | 0.2041 | 4.61 | 8 | 0.798 | 0.00 | 0.00 | Total between | 0.842 | 1 | 0.359 |
|  | NDSZ | 9 | -0.01 | 0.06 | 0.00 | -0.14 | 0.11 | -0.18 | 0.8605 | 5.37 | 8 | 0.717 | 0.00 | 0.00 |  |  |  |  |
| **Rostral**  **anterior**  **cingulate** | DSZ | 9 | -0.12 | 0.12 | 0.01 | -0.36 | 0.12 | -0.99 | 0.3217 | 13.61 | 8 | 0.092 | 41.22 | 0.05 | Total between | 0.327 | 1 | 0.567 |
|  | NDSZ | 9 | -0.03 | 0.10 | 0.01 | -0.22 | 0.16 | -0.33 | 0.7450 | 16.36 | 8 | 0.038 | 51.09 | 0.04 |  |  |  |  |
| **Rostral**  **middle**  **frontal** | DSZ | 9 | -0.14 | 0.11 | 0.01 | -0.35 | 0.07 | -1.33 | 0.1839 | 10.67 | 8 | 0.221 | 25.04 | 0.02 | Total between | 0.593 | 1 | 0.441 |
|  | NDSZ | 9 | -0.03 | 0.09 | 0.01 | -0.21 | 0.14 | -0.38 | 0.7052 | 14.04 | 8 | 0.081 | 43.03 | 0.03 |  |  |  |  |
| **Superior**  **frontal** | DSZ | 9 | -0.09 | 0.12 | 0.01 | -0.33 | 0.15 | -0.76 | 0.4500 | 13.42 | 8 | 0.098 | 40.38 | 0.05 | Total between | 0.238 | 1 | 0.626 |
|  | NDSZ | 9 | -0.01 | 0.13 | 0.02 | -0.25 | 0.24 | -0.05 | 0.9593 | 26.94 | 8 | 0.001 | 70.31 | 0.09 |  |  |  |  |
| **Superior**  **parietal** | DSZ | 9 | -0.06 | 0.09 | 0.01 | -0.24 | 0.12 | -0.66 | 0.5092 | 8.74 | 8 | 0.364 | 8.49 | 0.01 | Total between | 0.332 | 1 | 0.565 |
|  | NDSZ | 9 | 0.01 | 0.08 | 0.01 | -0.15 | 0.17 | 0.12 | 0.9067 | 11.32 | 8 | 0.184 | 29.30 | 0.02 |  |  |  |  |
| **Superior**  **temporal** | DSZ | 9 | -0.13 | 0.11 | 0.01 | -0.36 | 0.09 | -1.18 | 0.2391 | 11.76 | 8 | 0.162 | 31.95 | 0.03 | Total between | 0.106 | 1 | 0.744 |
|  | NDSZ | 9 | -0.09 | 0.10 | 0.01 | -0.27 | 0.10 | -0.89 | 0.3709 | 15.39 | 8 | 0.052 | 48.03 | 0.04 |  |  |  |  |
| **Supramarginal** | DSZ | 9 | -0.08 | 0.13 | 0.02 | -0.35 | 0.18 | -0.61 | 0.5424 | 16.14 | 8 | 0.040 | 50.43 | 0.07 | Total between | 0.447 | 1 | 0.504 |
|  | NDSZ | 9 | 0.03 | 0.09 | 0.01 | -0.16 | 0.21 | 0.30 | 0.7675 | 15.07 | 8 | 0.058 | 46.91 | 0.03 |  |  |  |  |
| **Temporal pole** | DSZ | 9 | -0.04 | 0.09 | 0.01 | -0.22 | 0.15 | -0.41 | 0.6832 | 9.03 | 8 | 0.340 | 11.36 | 0.01 | Total between | 0.015 | 1 | 0.901 |
|  | NDSZ | 9 | -0.02 | 0.07 | 0.00 | -0.16 | 0.11 | -0.34 | 0.7344 | 9.33 | 8 | 0.315 | 14.23 | 0.01 |  |  |  |  |
| **Transverse**  **temporal** | DSZ | 9 | -0.22 | 0.11 | 0.01 | -0.44 | 0.01 | -1.91 | 0.0563 | 12.06 | 8 | 0.149 | 33.64 | 0.04 | Total between | 0.515 | 1 | 0.473 |
|  | NDSZ | 9 | -0.11 | 0.09 | 0.01 | -0.29 | 0.07 | -1.22 | 0.2232 | 14.79 | 8 | 0.063 | 45.92 | 0.03 |  |  |  |  |
| **Total Surface** | DSZ | 9 | -0.24 | 0.14 | 0.02 | -0.51 | 0.03 | -1.73 | 0.0843 | 16.92 | 8 | 0.031 | 52.71 | 0.08 | Total between | 0.423 | 1 | 0.516 |
|  | NDSZ | 9 | -0.12 | 0.12 | 0.01 | -0.35 | 0.11 | -1.01 | 0.3111 | 23.69 | 8 | 0.003 | 66.24 | 0.08 |  |  |  |  |

**Legend:** *DSZ, Deficit Schizophrenia; NDSZ, Non Deficit Schizophrenia; Point estim, estimated standard difference; Std err, Standard Error; Var, Variance; df, degrees of freedom; Sqrd, Squared*

**Supplementary Table S11**. Regions excluded for significant heterogeneity in DSZ

| **Cortical**  **Regions** | **Group** | **Effect size and 95% confidence interval** | | | | | | **Test of null**  **(2-Tail)** | | **Heterogeneity** | | | | **Tau- Sqrd** |
| --- | --- | --- | --- | --- | --- | --- | --- | --- | --- | --- | --- | --- | --- | --- |
|  |  | **N** | **Point estim** | **Std err** | **Var** | **Low limit** | **Up limit** | **Z-value** | ***P-***  ***value*** | **Q-value** | **df** | ***P-value*** | **I-**  **sqrd** |  |
| **L. Fusiform** | DSZ | 9 | -0.43 | 0.18 | 0.03 | -0.77 | -0.08 | -2.41 | 0.0158 | 27.64 | 8 | 0.0005 | 71.05 | 0.18 |
| **L. Inferior temporal** | DSZ | 9 | -0.35 | 0.18 | 0.03 | -0.70 | -0.01 | -2.00 | 0.0458 | 27.14 | 8 | 0.0007 | 70.52 | 0.18 |
| **L. Insula** | DSZ | 9 | -0.38 | 0.16 | 0.03 | -0.69 | -0.06 | -2.34 | 0.0195 | 22.82 | 8 | 0.0036 | 64.95 | 0.14 |
| **L. Lateral orbitofrontal** | DSZ | 9 | -0.40 | 0.18 | 0.03 | -0.75 | -0.04 | -2.20 | 0.0279 | 28.64 | 8 | 0.0004 | 72.06 | 0.19 |
| **L. Middle temporal** | DSZ | 9 | -0.51 | 0.17 | 0.03 | -0.84 | -0.18 | -3.06 | 0.0022 | 23.46 | 8 | 0.0028 | 65.90 | 0.15 |
| **L. Rostral anterior cingulate** | DSZ | 9 | -0.35 | 0.18 | 0.03 | -0.70 | 0.00 | -1.97 | 0.0486 | 28.01 | 8 | 0.0005 | 71.43 | 0.18 |
| **L. Rostral middle frontal** | DSZ | 9 | -0.51 | 0.17 | 0.03 | -0.85 | -0.17 | -2.98 | 0.0029 | 26.28 | 8 | 0.0009 | 69.56 | 0.17 |
| **L. Superior frontal** | DSZ | 9 | -0.71 | 0.17 | 0.03 | -1.04 | -0.37 | -4.15 | <0.0001 | 25.65 | 8 | 0.0012 | 68.81 | 0.16 |
| **L. Superior temporal** | DSZ | 9 | -0.54 | 0.16 | 0.02 | -0.85 | -0.23 | -3.40 | 0.0007 | 20.73 | 8 | 0.0079 | 61.41 | 0.12 |
| **L. Mean thickness** | DSZ | 9 | -0.67 | 0.15 | 0.02 | -0.97 | -0.37 | -4.40 | <0.0001 | 20.42 | 8 | 0.0088 | 60.83 | 0.11 |
| **R. Fusiform** | DSZ | 9 | -0.54 | 0.16 | 0.03 | -0.85 | -0.23 | -3.38 | 0.0007 | 22.42 | 8 | 0.0042 | 64.32 | 0.12 |
| **R. Inferior temporal** | DSZ | 9 | -0.44 | 0.14 | 0.02 | -0.72 | -0.16 | -3.10 | 0.0019 | 17.41 | 8 | 0.0261 | 54.05 | 0.13 |
| **R. Lateral occipital** | DSZ | 9 | -0.34 | 0.16 | 0.02 | -0.65 | -0.04 | -2.21 | 0.0270 | 21.20 | 8 | 0.0066 | 62.26 | 0.12 |
| **R. Middle temporal** | DSZ | 9 | -0.49 | 0.13 | 0.02 | -0.75 | -0.23 | -3.66 | 0.0003 | 15.79 | 8 | 0.0454 | 49.35 | 0.07 |
| **R. Pars opercularis** | DSZ | 9 | -0.42 | 0.14 | 0.02 | -0.69 | -0.14 | -2.95 | 0.0032 | 17.60 | 8 | 0.0244 | 54.55 | 0.07 |
| **R. Postcentral** | DSZ | 9 | -0.46 | 0.15 | 0.02 | -0.75 | -0.17 | -3.11 | 0.0019 | 19.07 | 8 | 0.0145 | 58.05 | 0.07 |
| **R. Superior temporal** | DSZ | 9 | -0.52 | 0.15 | 0.02 | -0.80 | -0.23 | -3.55 | 0.0004 | 18.03 | 8 | 0.0210 | 55.64 | 0.10 |
| **R. Mean thickness** | DSZ | 9 | -0.62 | 0.15 | 0.02 | -0.90 | -0.33 | -4.26 | <0.0001 | 18.40 | 8 | 0.0184 | 56.52 | 0.12 |

**Legend:** *DSZ, Deficit Schizophrenia; L, Left; Point estim, estimated standard difference; R, Right; Std err, Standard Error; Var, Variance; df, degrees of freedom; Sqrd, Squared*

**Supplementary Table S12**. Regions excluded for significant heterogeneity in NDSZ

| **Cortical**  **Regions** | **Group** | **Effect size and 95% confidence interval** | | | | | | **Test of null**  **(2-Tail)** | | **Heterogeneity** | | | | |
| --- | --- | --- | --- | --- | --- | --- | --- | --- | --- | --- | --- | --- | --- | --- |
|  |  | **N** | **Point estim** | **Std err** | **Var** | **Low limit** | **Up limit** | **Z-value** | ***P-***  ***value*** | **Q-value** | **df** | ***P-value*** | **I-**  **sqrd** | **Tau- Sqrd** |
| **L. Bankssts** | NDSZ | 9 | -0.32 | 0.10 | 0.01 | -0.52 | -0.12 | -3.19 | 0.0014 | 16.91 | 8 | 0.0310 | 52.70 | 0.04 |
| **L. Fusiform** | NDSZ | 9 | -0.43 | 0.17 | 0.03 | -0.77 | -0.09 | -2.51 | 0.0122 | 50.62 | 8 | <0.0001 | 84.20 | 0.21 |
| **L. Inferior temporal** | NDSZ | 9 | -0.29 | 0.15 | 0.02 | -0.58 | 0.00 | -1.98 | 0.0475 | 36.55 | 8 | <0.0001 | 78.11 | 0.14 |
| **L. Insula** | NDSZ | 9 | -0.34 | 0.15 | 0.02 | -0.64 | -0.04 | -2.19 | 0.0284 | 39.92 | 8 | <0.0001 | 79.96 | 0.16 |
| **L. Isthmus cingulate** | NDSZ | 9 | -0.28 | 0.10 | 0.01 | -0.47 | -0.09 | -2.88 | 0.0040 | 16.45 | 8 | 0.0364 | 51.36 | 0.04 |
| **L. Lateral occipital** | NDSZ | 9 | -0.33 | 0.15 | 0.02 | -0.62 | -0.04 | -2.25 | 0.0244 | 35.97 | 8 | <0.0001 | 77.76 | 0.14 |
| **L. Lingual** | NDSZ | 9 | -0.29 | 0.14 | 0.02 | -0.56 | -0.01 | -2.05 | 0.0408 | 33.21 | 8 | <0.0001 | 75.91 | 0.12 |
| **L. Middle temporal** | NDSZ | 9 | -0.32 | 0.14 | 0.02 | -0.60 | -0.04 | -2.25 | 0.0246 | 33.85 | 8 | <0.0001 | 76.36 | 0.13 |
| **L. Para-hippocampal** | NDSZ | 9 | -0.28 | 0.10 | 0.01 | -0.47 | -0.08 | -2.82 | 0.0048 | 16.38 | 8 | 0.0372 | 51.17 | 0.04 |
| **L. Pars triangularis** | NDSZ | 9 | -0.31 | 0.11 | 0.01 | -0.52 | -0.09 | -2.73 | 0.0063 | 21.08 | 8 | 0.0069 | 62.05 | 0.06 |
| **L. Posterior cingulate** | NDSZ | 9 | -0.34 | 0.10 | 0.01 | -0.55 | -0.14 | -3.29 | 0.0010 | 18.33 | 8 | 0.0189 | 56.37 | 0.05 |
| **L. Precuneus** | NDSZ | 9 | -0.26 | 0.12 | 0.01 | -0.49 | -0.02 | -2.17 | 0.0301 | 24.18 | 8 | 0.0021 | 66.92 | 0.08 |
| **L. Rostral middle frontal** | NDSZ | 9 | -0.38 | 0.12 | 0.01 | -0.62 | -0.14 | -3.13 | 0.0017 | 24.96 | 8 | 0.0016 | 67.95 | 0.08 |
| **L. Superior frontal** | NDSZ | 9 | -0.56 | 0.10 | 0.01 | -0.76 | -0.36 | -5.45 | <0.0001 | 17.59 | 8 | 0.0245 | 54.53 | 0.05 |
| **L. Superior temporal** | NDSZ | 9 | -0.41 | 0.12 | 0.01 | -0.64 | -0.18 | -3.52 | 0.0004 | 21.97 | 8 | 0.0050 | 63.58 | 0.07 |
| **L. Temporal pole** | NDSZ | 9 | -0.24 | 0.10 | 0.01 | -0.43 | -0.04 | -2.38 | 0.0174 | 16.79 | 8 | 0.0324 | 52.36 | 0.04 |
| **L. Mean thickness** | NDSZ | 9 | -0.50 | 0.13 | 0.02 | -0.76 | -0.24 | -3.74 | 0.0002 | 29.46 | 8 | 0.0003 | 72.84 | 0.11 |
| **R. Fusiform** | NDSZ | 9 | -0.41 | 0.14 | 0.02 | -0.69 | -0.14 | -2.94 | 0.0033 | 33.20 | 8 | <0.0001 | 75.91 | 0.12 |
| **R. Inferior parietal** | NDSZ | 9 | -0.38 | 0.11 | 0.01 | -0.59 | -0.18 | -3.63 | 0.0003 | 18.97 | 8 | 0.0150 | 57.82 | 0.05 |
| **R. Inferior temporal** | NDSZ | 9 | -0.38 | 0.14 | 0.02 | -0.66 | -0.09 | -2.62 | 0.0088 | 33.99 | 8 | <0.0001 | 76.46 | 0.13 |
| **R. Isthmus cingulate** | NDSZ | 9 | -0.32 | 0.10 | 0.01 | -0.52 | -0.12 | -3.08 | 0.0020 | 18.25 | 8 | 0.0194 | 56.18 | 0.05 |
| **R. Lateral occipital** | NDSZ | 8 | -0.31 | 0.14 | 0.02 | -0.59 | -0.03 | -2.18 | 0.0289 | 31.72 | 7 | <0.0001 | 77.93 | 0.12 |
| **R. Lingual** | NDSZ | 8 | -0.31 | 0.12 | 0.01 | -0.54 | -0.08 | -2.69 | 0.0072 | 20.89 | 7 | 0.0039 | 66.50 | 0.07 |
| **R. Middle temporal** | NDSZ | 9 | -0.32 | 0.12 | 0.01 | -0.54 | -0.09 | -2.73 | 0.0064 | 22.29 | 8 | 0.0044 | 64.11 | 0.07 |
| **R. Pars opercularis** | NDSZ | 9 | -0.25 | 0.12 | 0.01 | -0.48 | -0.02 | -2.12 | 0.0340 | 23.02 | 8 | 0.0033 | 65.25 | 0.07 |
| **R. Pars orbitalis** | NDSZ | 9 | -0.25 | 0.10 | 0.01 | -0.44 | -0.06 | -2.61 | 0.0089 | 15.78 | 8 | 0.0457 | 49.29 | 0.04 |
| **R. Postcentral** | NDSZ | 9 | -0.29 | 0.12 | 0.01 | -0.51 | -0.06 | -2.50 | 0.0126 | 22.37 | 8 | 0.0043 | 64.24 | 0.07 |
| **R. Posterior cingulate** | NDSZ | 9 | -0.25 | 0.11 | 0.01 | -0.47 | -0.04 | -2.31 | 0.0206 | 20.56 | 8 | 0.0084 | 61.10 | 0.06 |
| **R. Precentral** | NDSZ | 9 | -0.25 | 0.11 | 0.01 | -0.46 | -0.04 | -2.38 | 0.0172 | 18.84 | 8 | 0.0157 | 57.54 | 0.05 |
| **R. Precuneus** | NDSZ | 9 | -0.24 | 0.11 | 0.01 | -0.45 | -0.03 | -2.23 | 0.0259 | 19.37 | 8 | 0.0130 | 58.70 | 0.05 |
| **R. Rostral middle frontal** | NDSZ | 9 | -0.20 | 0.10 | 0.01 | -0.39 | -0.01 | -2.10 | 0.0361 | 16.11 | 8 | 0.0408 | 50.35 | 0.04 |
| **R. Superior frontal** | NDSZ | 9 | -0.42 | 0.10 | 0.01 | -0.62 | -0.22 | -4.08 | <0.0001 | 17.93 | 8 | 0.0218 | 55.37 | 0.05 |
| **R. Superior temporal** | NDSZ | 9 | -0.34 | 0.13 | 0.02 | -0.60 | -0.09 | -2.63 | 0.0086 | 27.75 | 8 | 0.0005 | 71.18 | 0.10 |
| **R. Supramarginal** | NDSZ | 9 | -0.48 | 0.10 | 0.01 | -0.67 | -0.29 | -4.97 | <0.0001 | 15.80 | 8 | 0.0453 | 49.37 | 0.04 |
| **R. Temporal pole** | NDSZ | 9 | -0.24 | 0.11 | 0.01 | -0.45 | -0.02 | -2.16 | 0.0309 | 20.69 | 8 | 0.0080 | 61.34 | 0.06 |
| **R. Mean thickness** | NDSZ | 9 | -0.44 | 0.14 | 0.02 | -0.71 | -0.16 | -3.15 | 0.0016 | 32.49 | 8 | <0.0001 | 75.38 | 0.12 |

**Legend:** *L, Left; NDSZ, Non Deficit Schizophrenia; Point estim, estimated standard difference; R, Right; Std err, Standard Error; Var, Variance; df, degrees of freedom; Sqrd, Squared*
